# Supplementary material for: Different molecular recognition by three domains of the full-length GRB2 to SOS1 proline-rich motifs and EGFR phosphorylated sites
Source: Chem Sci. 2024 Sep 12;15(38):15858–72. doi: 10.1039/d4sc02656j (PMC11391413; doi:10.1039/d4sc02656j)
Supplement: SC-015-D4SC02656J-s001 [file SC-015-D4SC02656J-s001.pdf]

Supplementary Information (SI) for Chemical Science.
This journal is © The Royal Society of Chemistry 2024

Supporting Information for:

Different molecular recognition by three domains of the full-length GRB2 to SOS1 proline-rich motifs and EGFR phosphorylated sites

Keita Tateno[a,+], Takami Ando[a,+], Maako Tabata[a], Haruka Sugasawa[a], Toshifumi Hayashi[a], Sanya Yu[a], Sayeesh PM[a], Kohsuke Inomata[a], Tsutomu Mikawa[b], Yuji Sugita[b], Yutaka Ito[a*] and Teppei Ikeya[a*]

[a]Department of Chemistry, Graduate School of Science, Tokyo Metropolitan University, 1-1 Minamiosawa, Hachioji, Tokyo 192-0397, Japan; [b]RIKEN Center for Biosystems Dynamics Research, RIKEN, 1-7-22 Suehiro-cho, Tsurumi-ku, Yokohama 230-0045, Japan; [c]Theoretical Molecular Science Laboratory, RIKEN Cluster for Pioneering Research, Wako, Saitama 351-0198, Japan; [d]Laboratory for Biomolecular Function Simulation, RIKEN Center for Biosystems Dynamics Research, Kobe, Hyogo 650-0047, Japan; [e]Computational Biophysics Research Team, RIKEN Center for Computational Science, Kobe, Hyogo 650-0047, Japan

[†] These authors contributed equally to this work

[*] To whom correspondence should be addressed. E-mail: T.I and Y.I (tikeya@tmu.ac.jp and ito-yutaka@tmu.ac.jp)

Contents

1. **Supplementary Figure S1.** Domain and three dimensional (3D) structures of GRB2 and SOS1, and schematic illustration of LLPS.
2. **Supplementary Figure S2.** Overlays of 2D ^{1}H-^{15}N HSQC spectra of ^{15}N-uniformly and amino-acid-selectively labelled samples.
3. **Supplementary Figure S3.** Backbone resonance assignments of GRB2.
4. **Supplementary Figure S4.** Table summarising the backbone and side-chain resonance assignments of GRB2.
5. **Supplementary Figure S5.** Overlays of 2D ^{1}H-^{15}N HSQC spectra of the full-length GRB2 and each isolated domain.
6. **Supplementary Figure S6.** Overlays of 2D ^{1}H-^{15}N HSQC spectra from multipoint titrations of ^{15}N-labelled GRB2 with SOS-S5 PRM.
7. **Supplementary Figure S7.** Overlays of 2D ^{1}H-^{15}N HSQC spectra from multipoint titrations of ^{15}N-labelled GRB2 with SOS-S9 PRM.
8. **Supplementary Figure S8.** Overlays of 2D ^{1}H-^{15}N HSQC spectra from multipoint titrations of ^{15}N-labelled GRB2 with SOS-S10 PRM.
9. **Supplementary Figure S9.** Plots of relative change in chemical shift perturbation (CSP) of backbone $^{1}H^{N}$ and ^{15}N nuclei of GRB2 upon the titration with S4, S5, S9, and S10 PRMs.
10. **Supplementary Figure S10.** Residual errors after the bootstrap calculations.
11. **Supplementary Figure S11.** Chemical shift perturbation profile mapped on each residue, against the concentration of ligand with SOS S4 PRM.
12. **Supplementary Figure S12.** Chemical shift perturbation profile mapped on each residue, against the concentration of ligand with SOS S5 PRM.
13. **Supplementary Figure S13.** Chemical shift perturbation profile mapped on each residue, against the concentration of ligand with SOS S9 PRM.
14. **Supplementary Figure S14.** Chemical shift perturbation profile mapped on each residue, against the concentration of ligand with SOS S10 PRM.
15. **Supplementary Figure S15.** ITC analysis for the binding of the full length GRB2 to S2,

S4, S5, S6, S8 S9, and S10 PRMs..

16. **Supplementary Figure S16.** O ;verlays of 2D ^{1}H-^{15}N HSQC spectra from multipoint titrations of ^{15}N-labelled GRB2 exclusively with the EGFR phosphorylated peptide.
17. **Supplementary Figure S17.** Overlays of 2D ^{1}H-^{15}N HSQC spectra from multipoint titrations of ^{15}N-labelled GRB2 with the EGFR phosphorylated peptide, in the presence of S4 PRM.
18. **Supplementary Table S1** Dissociation constants derived from ITC analysis for the full length GRB2 and isolated SH3 domains against SOS1 PRMs.

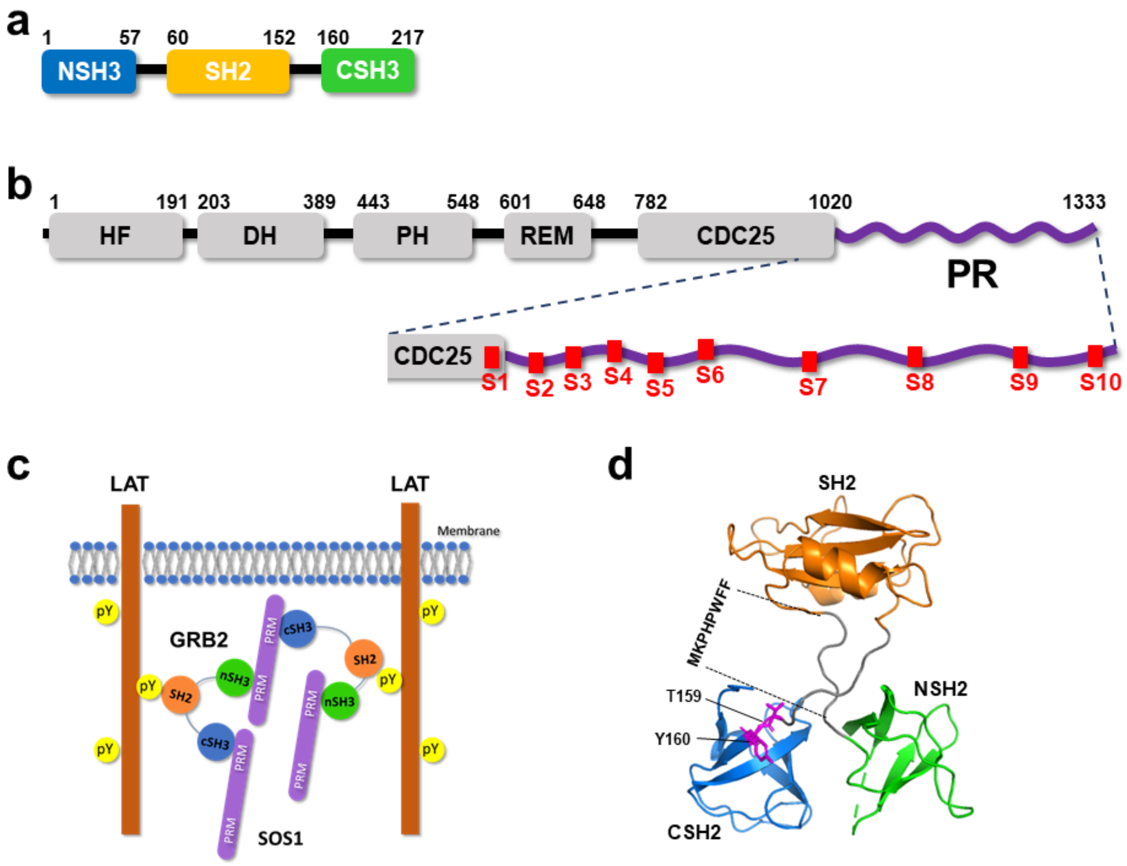

Figure S1. Domain and three dimensional (3D) structures of GRB2 and SOS1, and schematic illustration of LLPS. (a) the domain structure of GRB2. (b) the domain structure of SOS1 with the positions of proline-rich motifs, SOS1 S1-S10, indicated by red boxes. (c) Schematic illustration of the considered multivalent interactions among GRB2, SOS1 and LAT in liquid-liquid phase separation (LLPS), proposed in the previous reports. (d) The crystal structure of GRB2.

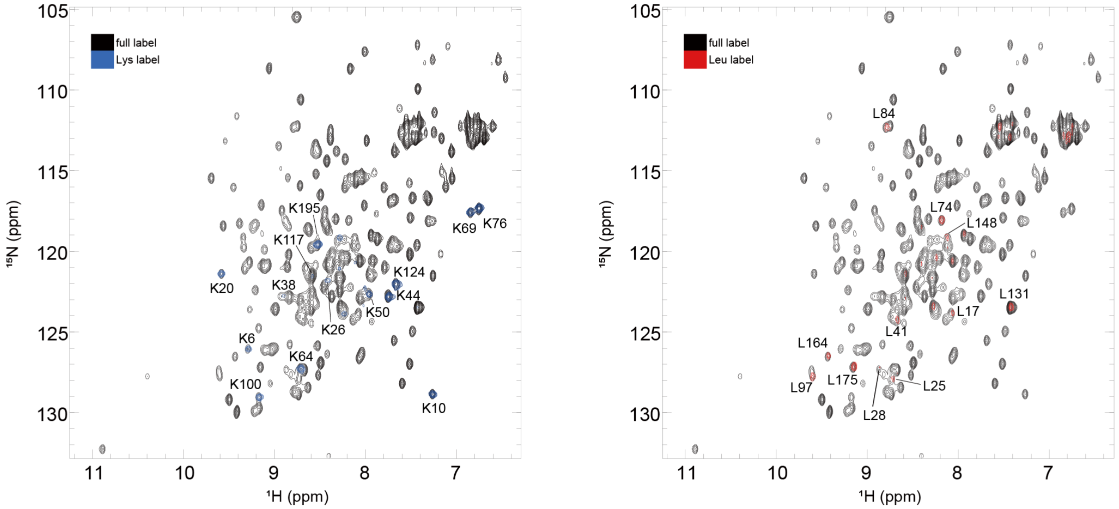

Figure S2. Overlays of 2D ^{1}H-^{15}N HSQC spectra of ^{15}N-uniformly and amino-acid-selectively labelled samples. The spectra of lysine- (left) and leucine- (right) selectively labelled GRB2 were overlaid onto those of the ^{15}N-uniformly labelled samples.

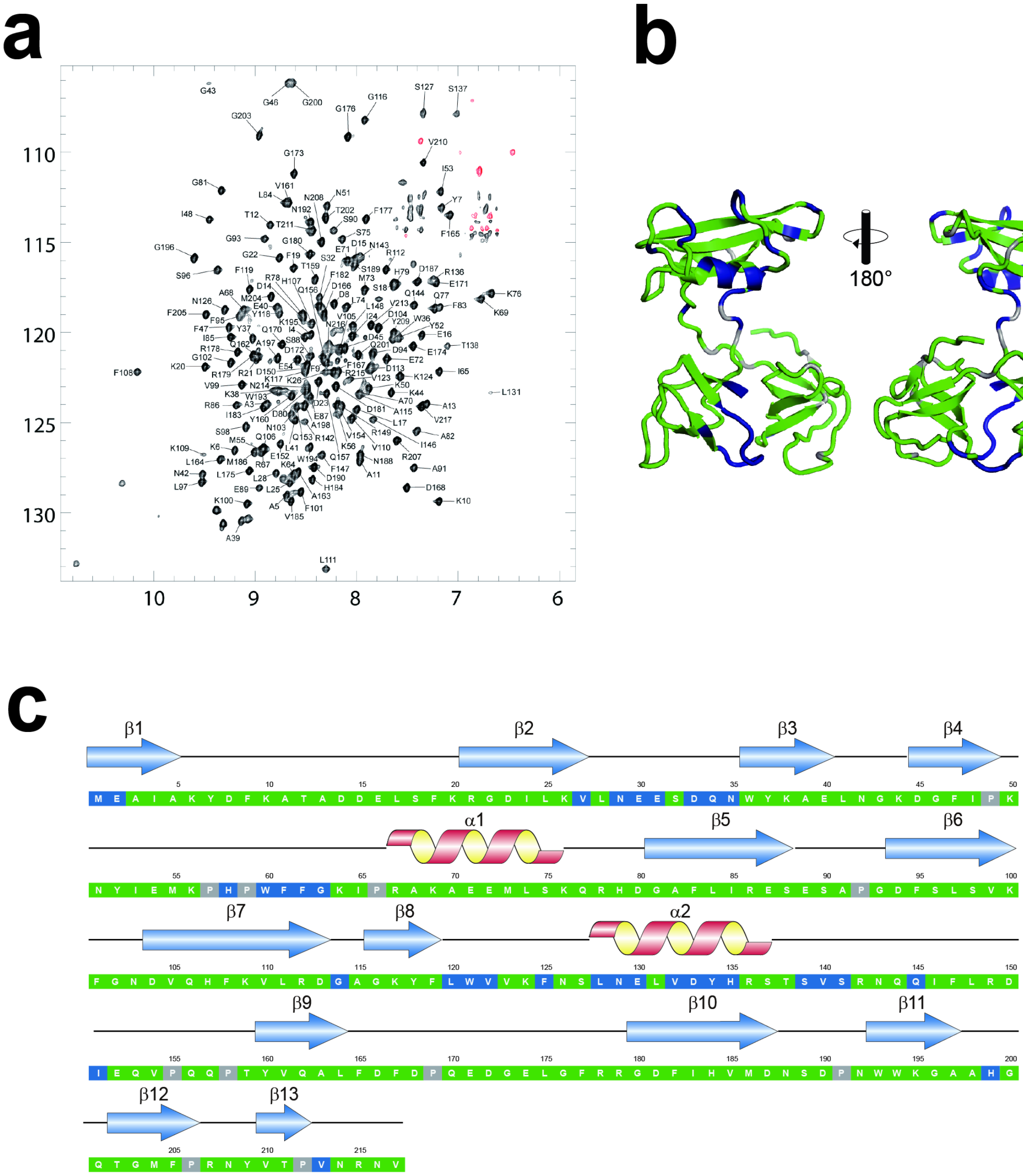

Figure S3. Backbone resonance assignments of GRB2. (a) 2D ^{1}H-^{15}N HSQC spectrum and backbone resonance assignments of GRB2. Positive and negative signals (aliased on the ^{15}N axis) are color-coded in black and red, respectively. (b) The backbone resonance assignments are mapped onto the crystal structure of GRB2. Assigned and unassigned residues are colour-coded in green and blue, respectively. (c) The backbone resonance assignments aligned with

secondary structures. Assigned, unassigned, and proline residues are colour-coded in green, blue, and grey, respectively.

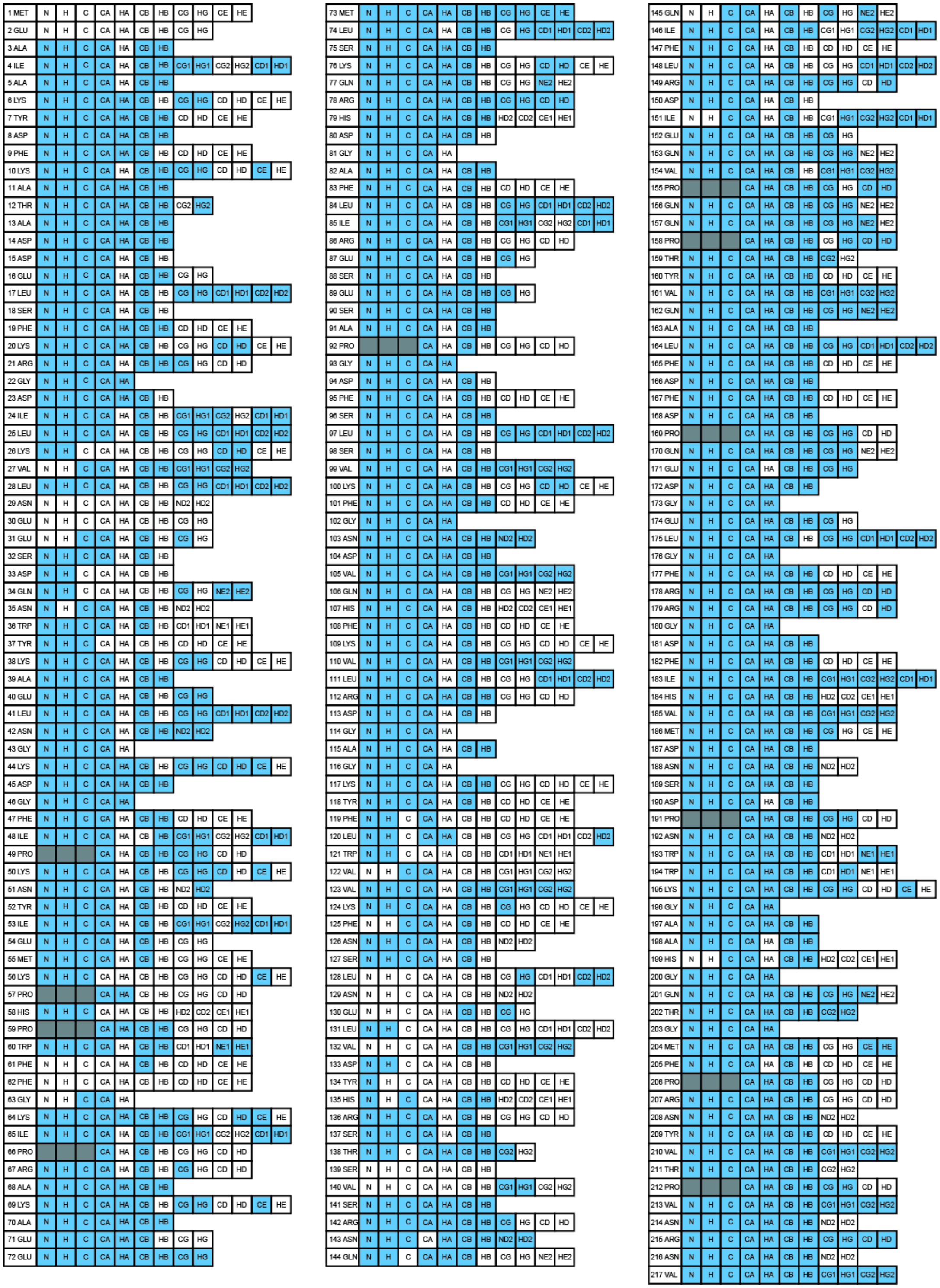

Figure S4. Table summarising the backbone and side-chain resonance assignments of GRB2. Blue and white columns indicate assigned and unassigned atoms, respectively.

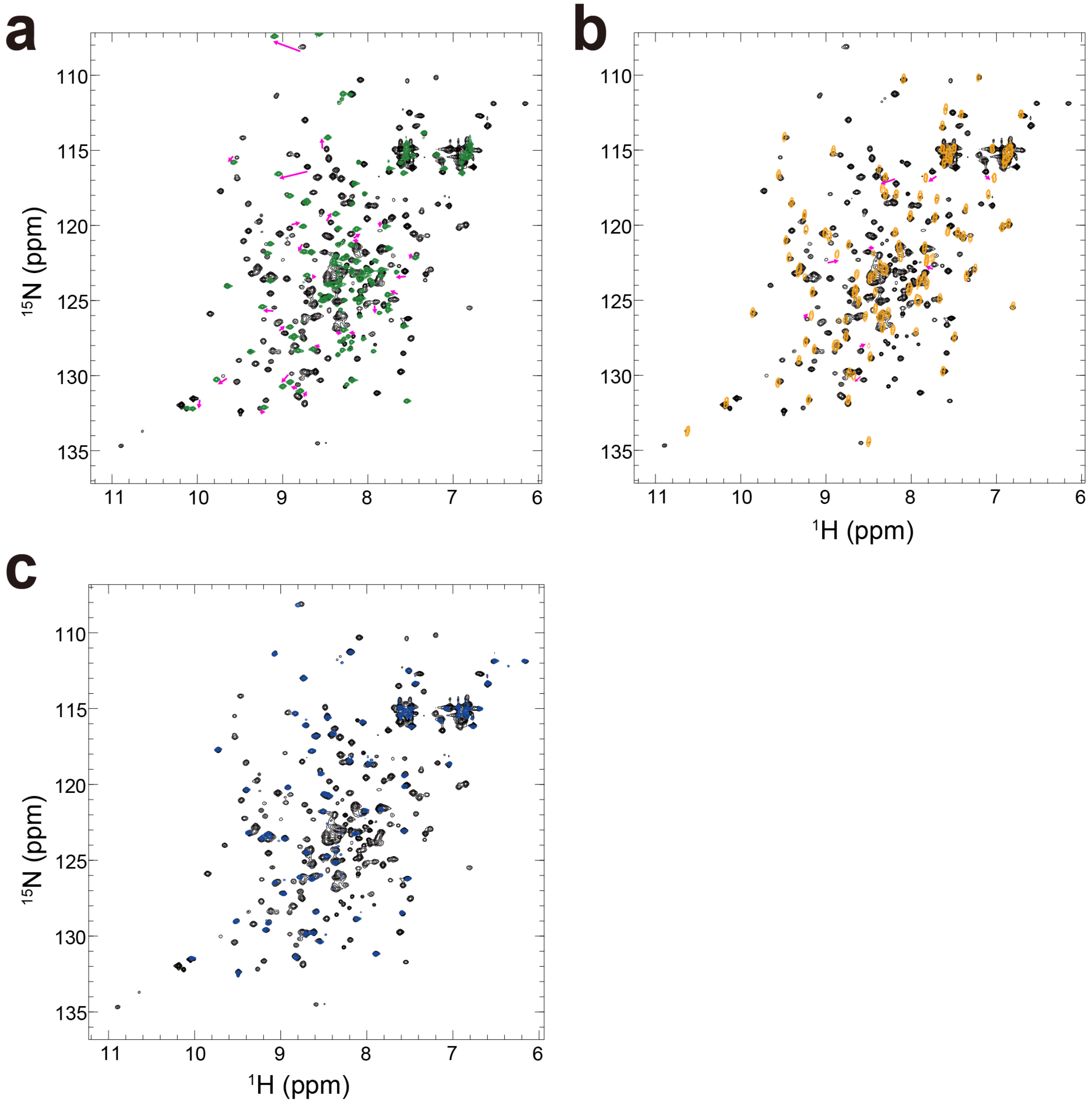

Figure S5. Overlays of 2D ^{1}H-^{15}N HSQC spectra of the full-length GRB2 and each isolated domain. The black spectra indicate the full-length GRB2, and green, orange, and blue represent isolated NSH3, SH2, CSH3, respectively. The red arrows indicate representative peaks that significant chemical shift changes were observed.

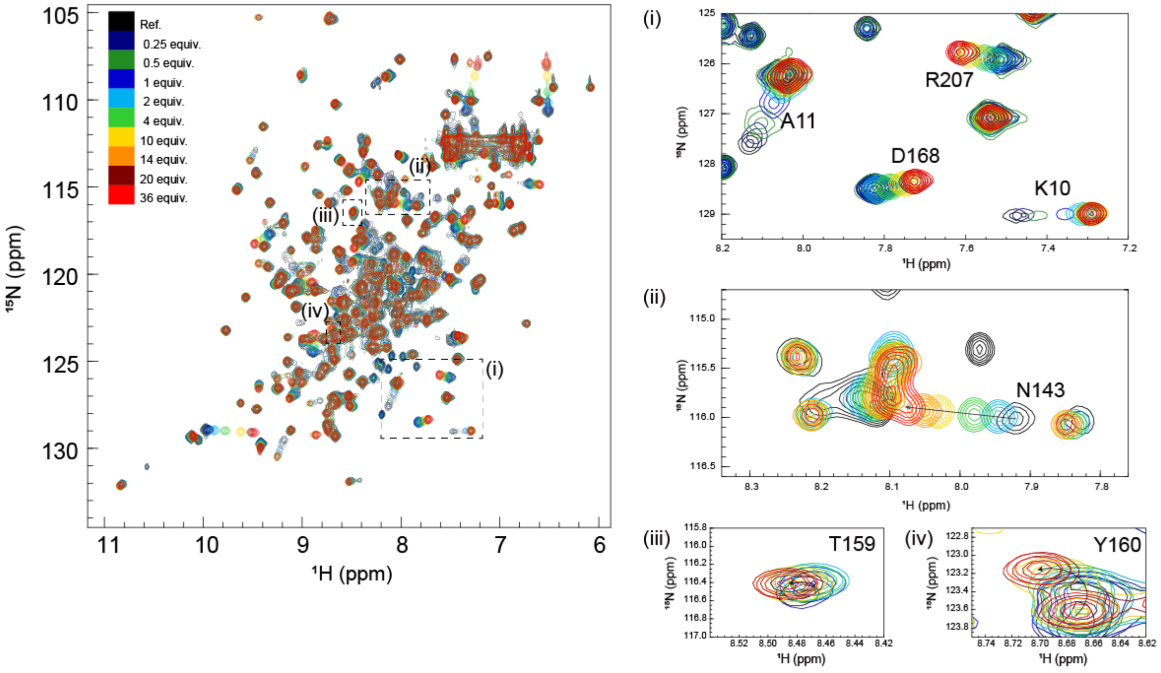

Figure S6. Overlays of 2D ^{1}H-^{15}N HSQC spectra from multipoint titrations of ^{15}N-labelled GRB2 with SOS-S5 PRM (DSPPAOPPRQPT). The peptide concentration was increased stepwise (the protein: peptide molar ratio of 1:0.25, 1:0.5, 1:1, 1:2, 1:4, 1:10, 1:14, 1:20, and 1:36). In this figure, the colour codes of ^{1}H-^{15}N correlation cross-peaks at each titration point, showing the molar ratio of GRB2: SOS-S5, are as follows: black (1:0); dark blue (1:0.25); dark green (1:0.5); blue (1:1); cyan (1:2); green (1:4); yellow (1:10); orange (1:14); dark red (1:20), red (1:36). Cross peaks that showed large chemical shift changes were annotated.

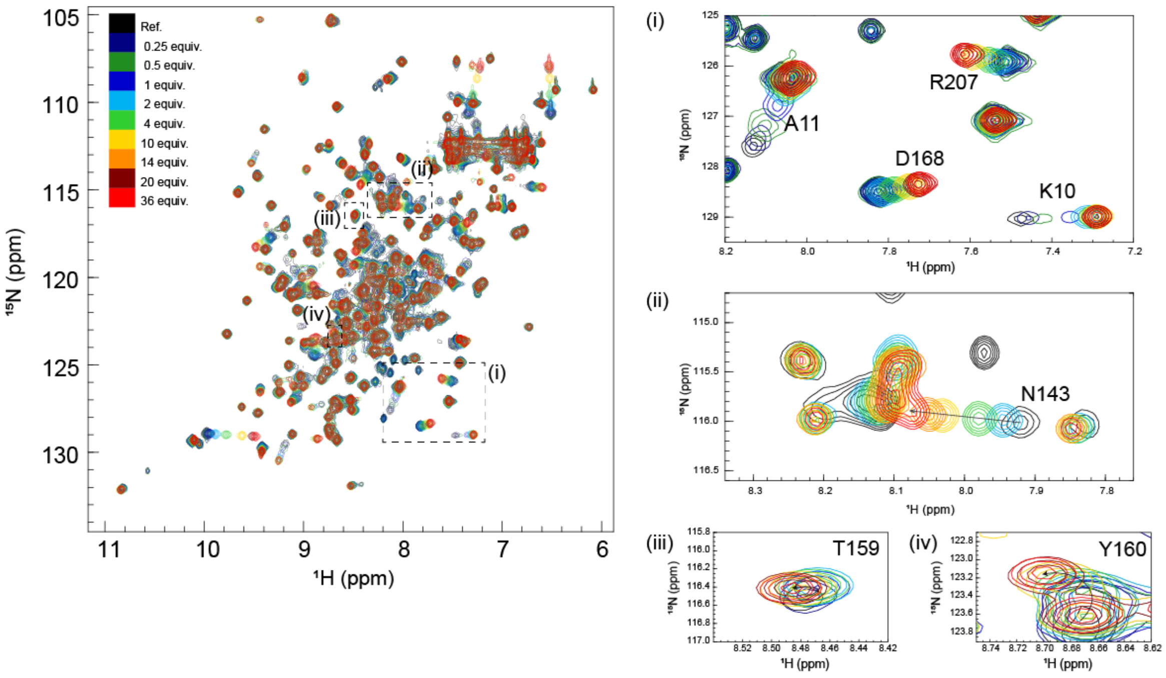

Figure S7. Overlays of 2D ^{1}H-^{15}N HSQC spectra from multipoint titrations of ^{15}N-labelled GRB2 with SOS-S9 PRM (IAGPPVPPRQST). The peptide concentration was increased stepwise (the protein: peptide molar ratio of 1:0.25, 1:0.5, 1:1, 1:2, 1:4, 1:10, 1:14, 1:20, and 1:36). In this figure, the colour codes of ^{1}H-^{15}N correlation cross-peaks at each titration point, showing the molar ratio of GRB2: SOS-S9, are as follows: black (1:0); dark blue (1:0.25); dark green (1:0.5); blue (1:1); cyan (1:2); green (1:4); yellow (1:10); orange (1:14); dark red (1:20), red (1:36). Cross peaks that showed large chemical shift changes were annotated.

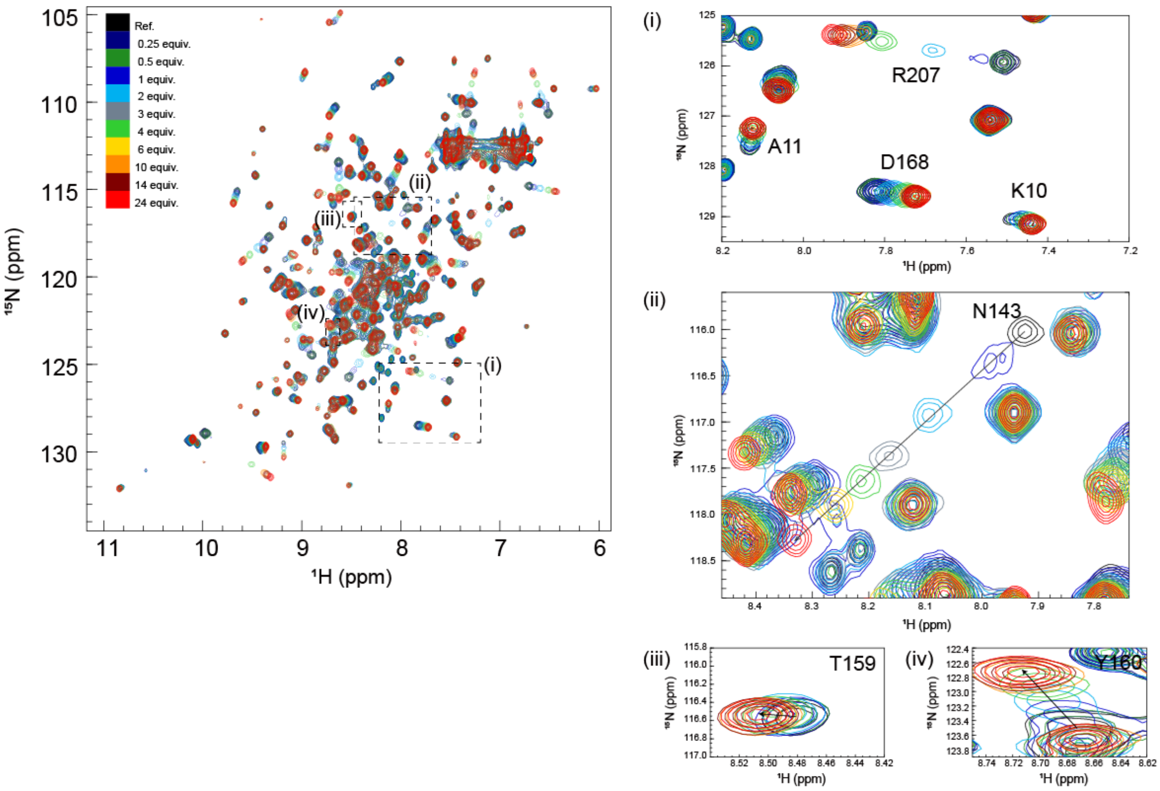

Figure S8. Overlays of 2D ^{1}H-^{15}N HSQC spectra from multipoint titrations of ^{15}N-labelled GRB2 with SOS-S10 PRM (PKLPPKTYKREH). The peptide concentration was increased stepwise (the protein: peptide molar ratio of 1:0.25, 1:0.5, 1:1, 1:2, 1:3, 1:4, 1:6, 1:10, 1:14, and 1:24). In this figure, the colour codes of ^{1}H-^{15}N correlation cross-peaks at each titration point, showing the molar ratio of GRB2: SOS-S10, are as follows: black (1:0); dark blue (1:0.25); dark green (1:0.5); blue (1:1); cyan (1:2); grey (1:3), green (1:4); yellow (1:6); orange (1:10); dark red (1:14), red (1:24). Cross peaks that showed large chemical shift changes were annotated.

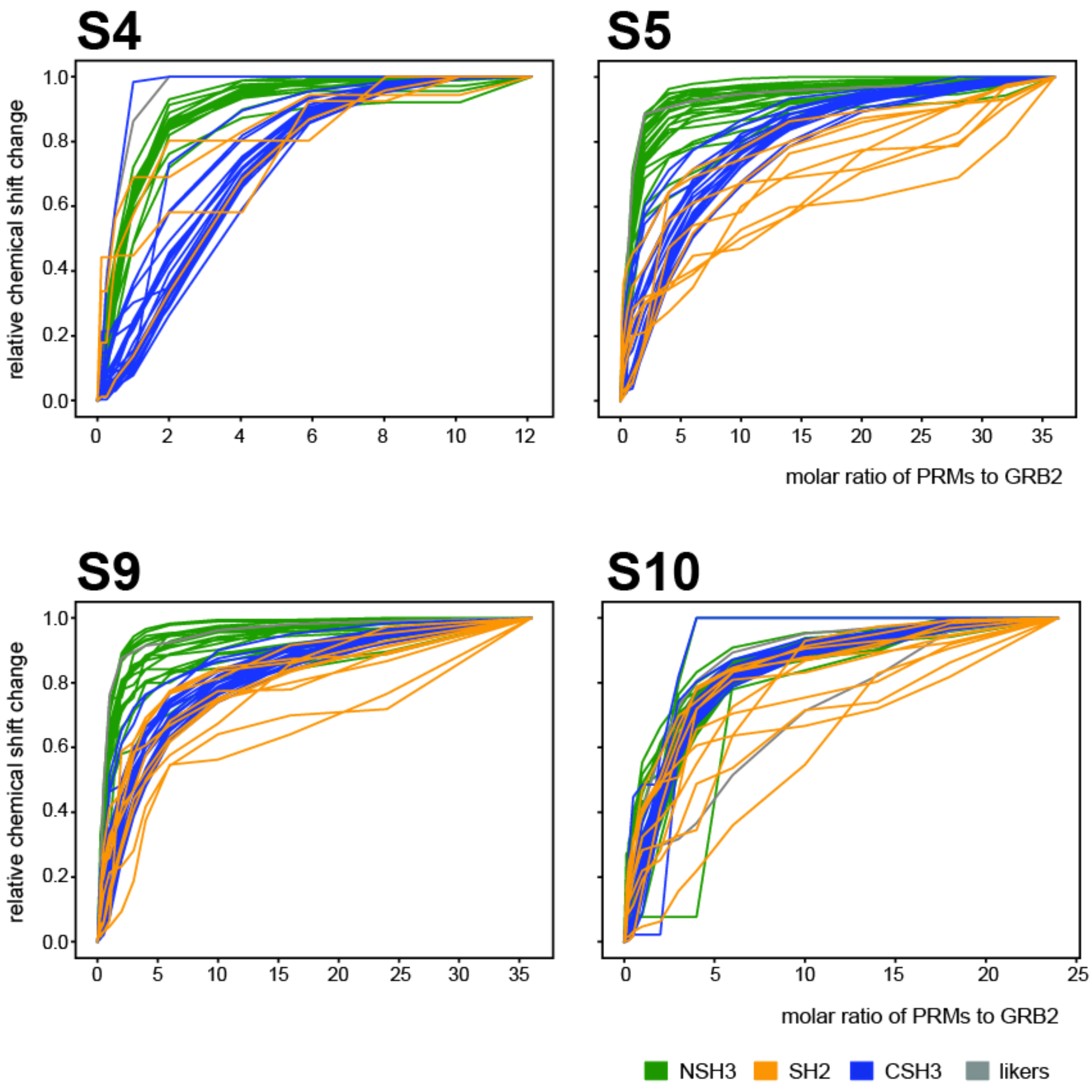

Figure S9. Plots of relative change in chemical shift perturbation (CSP) of backbone $^{1}H^{N}$ and ^{15}N nuclei of GRB2 upon the titration with S4, S5, S9, and S10 PRMs. A line represents the relative CSP beyond 0.06 ppm for a residue. Lines are colour-coded for each domain and linker.

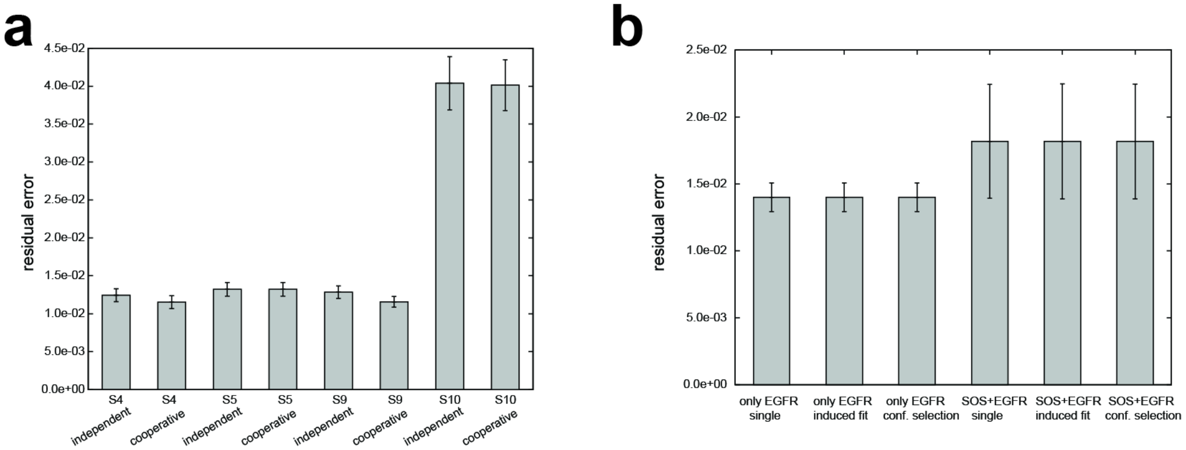

Figure S10. Residual errors after the bootstrap calculations. Residual errors after the bootstrap calculations with the CSPs of the S4, S5, S9, and S10 PRMs titration experiments for two-independent and two-cooperative binding models. residual errors after the bootstrap calculations for two-independent and two-cooperative binding models (right)

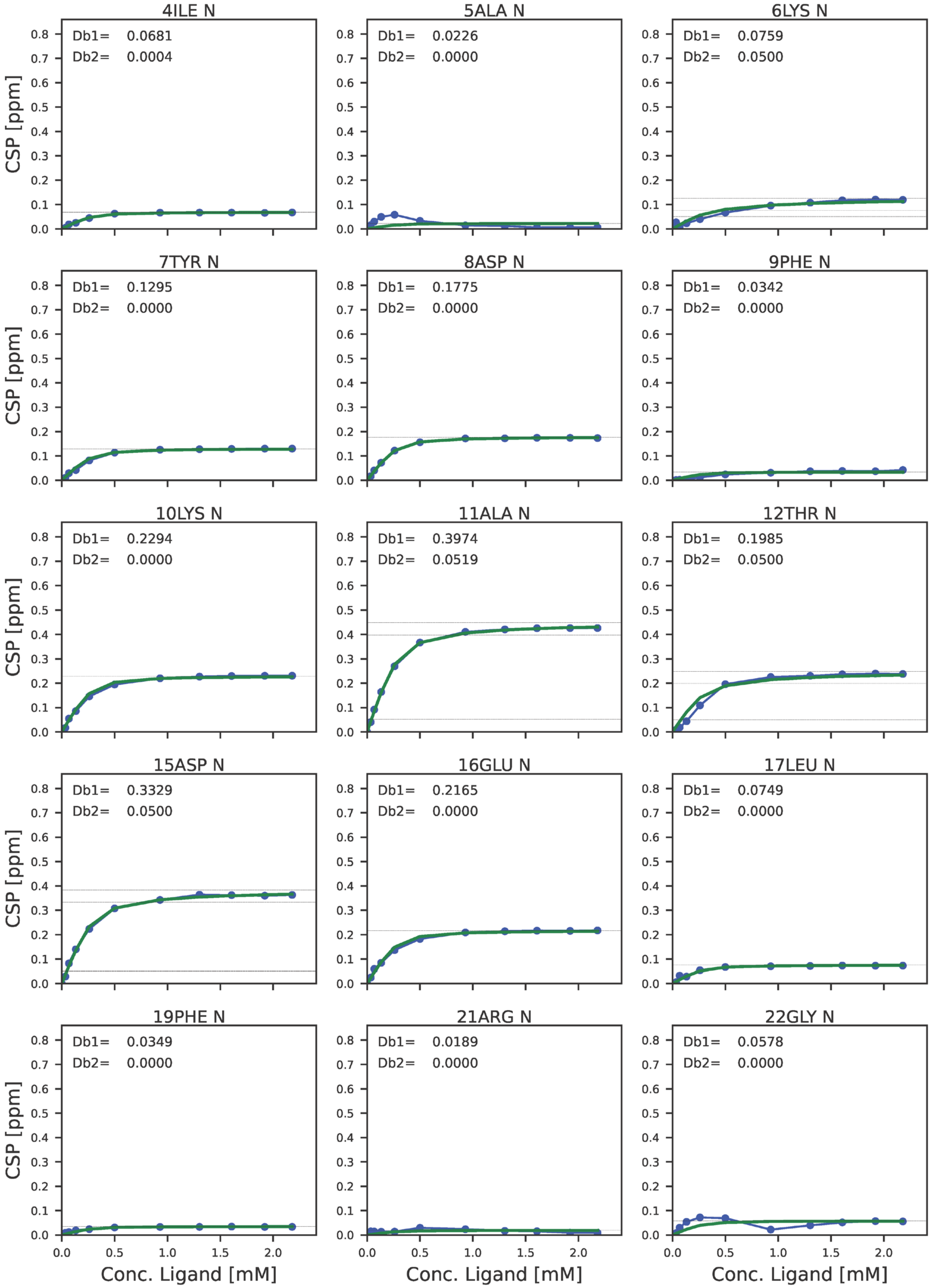
4ILE N
Db1= 0.0681
Db2= 0.0004
5ALA N
Db1= 0.0226
Db2= 0.0000
6LYS N
Db1= 0.0759
Db2= 0.0500
7TYR N
Db1= 0.1295
Db2= 0.0000
8ASP N
Db1= 0.1775
Db2= 0.0000
9PHE N
Db1= 0.0342
Db2= 0.0000
10LYS N
Db1= 0.2294
Db2= 0.0000
11ALA N
Db1= 0.3974
Db2= 0.0519
12THR N
Db1= 0.1985
Db2= 0.0500
15ASP N
Db1= 0.3329
Db2= 0.0500
16GLU N
Db1= 0.2165
Db2= 0.0000
17LEU N
Db1= 0.0749
Db2= 0.0000
19PHE N
Db1= 0.0349
Db2= 0.0000
21ARG N
Db1= 0.0189
Db2= 0.0000
22GLY N
Db1= 0.0578
Db2= 0.0000
CSP [ppm]
Conc. Ligand [mM]

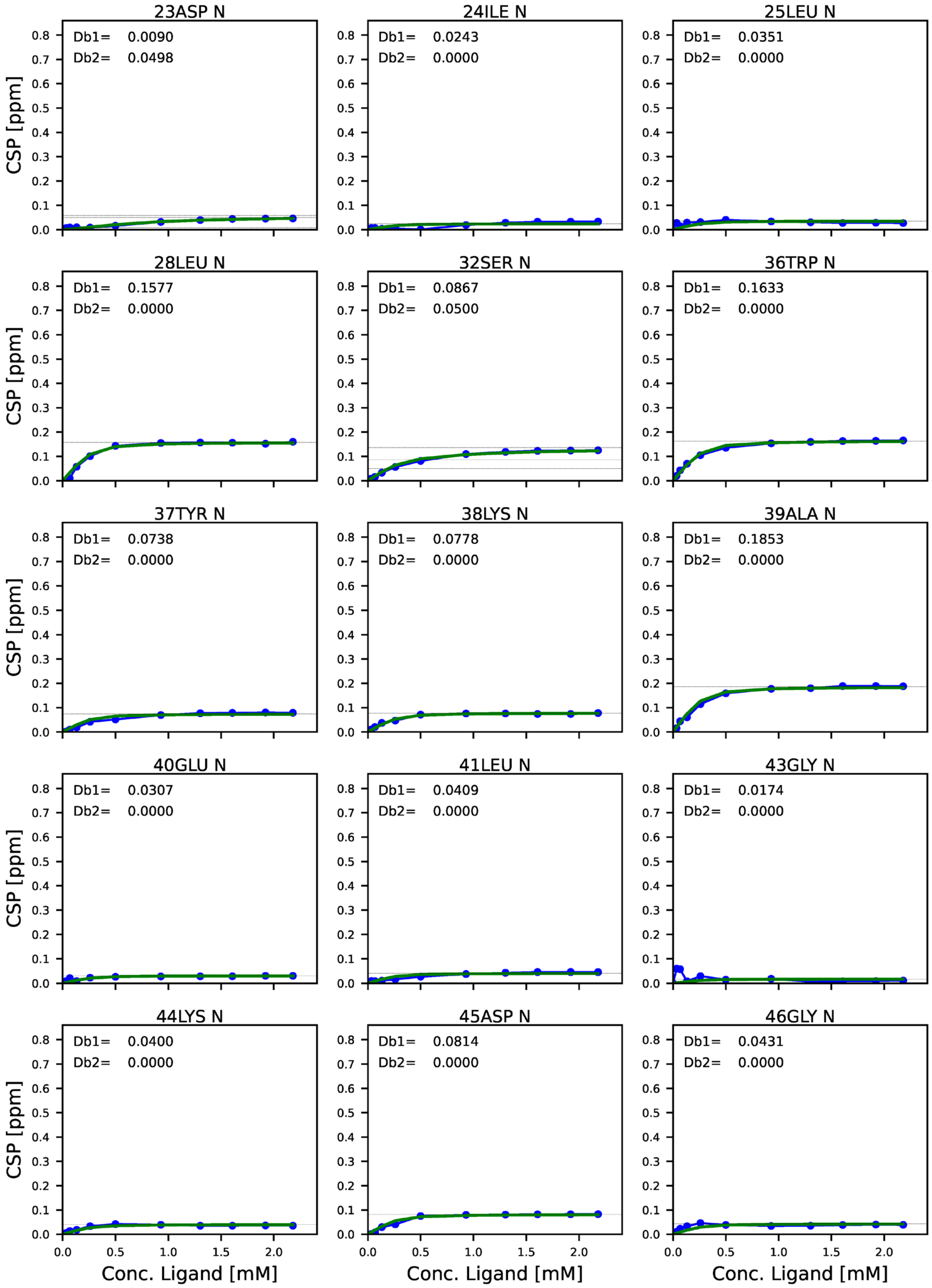

23ASP N
Db1= 0.0090
Db2= 0.0498
24ILE N
Db1= 0.0243
Db2= 0.0000
25LEU N
Db1= 0.0351
Db2= 0.0000
28LEU N
Db1= 0.1577
Db2= 0.0000
32SER N
Db1= 0.0867
Db2= 0.0500
36TRP N
Db1= 0.1633
Db2= 0.0000
37TYR N
Db1= 0.0738
Db2= 0.0000
38LYS N
Db1= 0.0778
Db2= 0.0000
39ALA N
Db1= 0.1853
Db2= 0.0000
40GLU N
Db1= 0.0307
Db2= 0.0000
41LEU N
Db1= 0.0409
Db2= 0.0000
43GLY N
Db1= 0.0174
Db2= 0.0000
44LYS N
Db1= 0.0400
Db2= 0.0000
45ASP N
Db1= 0.0814
Db2= 0.0000
46GLY N
Db1= 0.0431
Db2= 0.0000
CSP [ppm]
Conc. Ligand [mM]

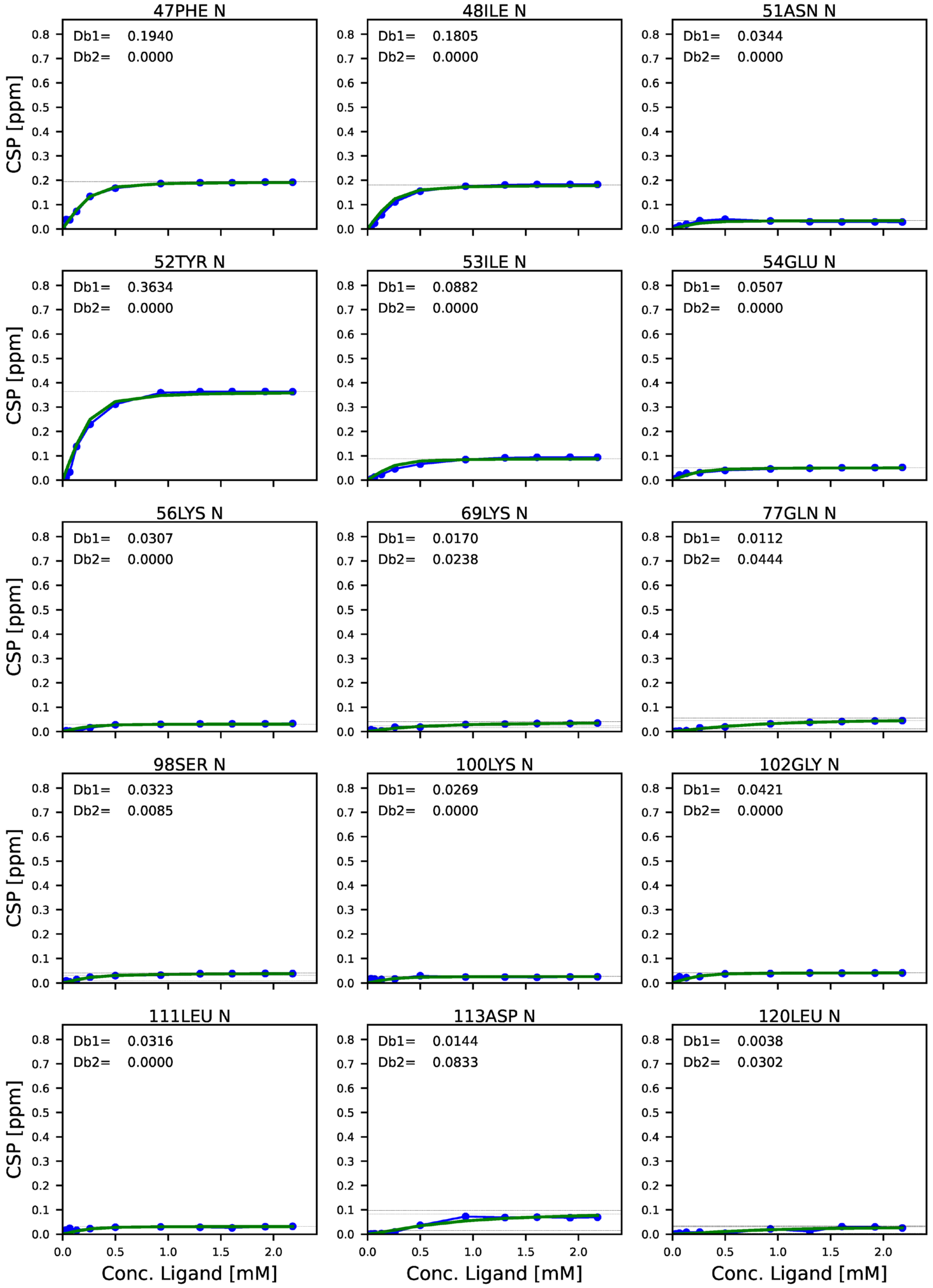
47PHE N
Db1= 0.1940
Db2= 0.0000
48ILE N
Db1= 0.1805
Db2= 0.0000
51ASN N
Db1= 0.0344
Db2= 0.0000
52TYR N
Db1= 0.3634
Db2= 0.0000
53ILE N
Db1= 0.0882
Db2= 0.0000
54GLU N
Db1= 0.0507
Db2= 0.0000
56LYS N
Db1= 0.0307
Db2= 0.0000
69LYS N
Db1= 0.0170
Db2= 0.0238
77GLN N
Db1= 0.0112
Db2= 0.0444
98SER N
Db1= 0.0323
Db2= 0.0085
100LYS N
Db1= 0.0269
Db2= 0.0000
102GLY N
Db1= 0.0421
Db2= 0.0000
111LEU N
Db1= 0.0316
Db2= 0.0000
113ASP N
Db1= 0.0144
Db2= 0.0833
120LEU N
Db1= 0.0038
Db2= 0.0302
CSP [ppm]
Conc. Ligand [mM]

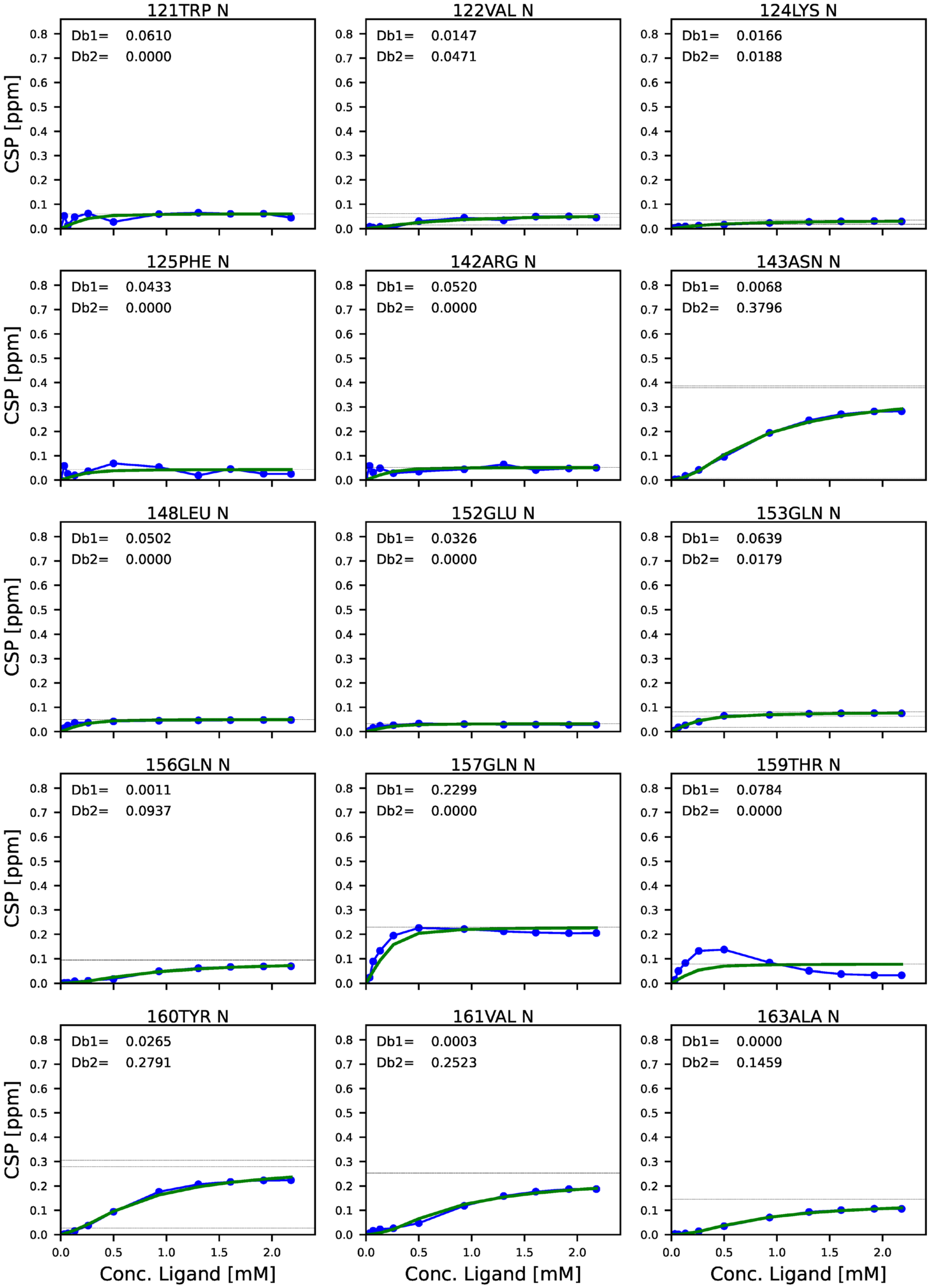
121TRP N
Db1= 0.0610
Db2= 0.0000
122VAL N
Db1= 0.0147
Db2= 0.0471
124LYS N
Db1= 0.0166
Db2= 0.0188
125PHE N
Db1= 0.0433
Db2= 0.0000
142ARG N
Db1= 0.0520
Db2= 0.0000
143ASN N
Db1= 0.0068
Db2= 0.3796
148LEU N
Db1= 0.0502
Db2= 0.0000
152GLU N
Db1= 0.0326
Db2= 0.0000
153GLN N
Db1= 0.0639
Db2= 0.0179
156GLN N
Db1= 0.0011
Db2= 0.0937
157GLN N
Db1= 0.2299
Db2= 0.0000
159THR N
Db1= 0.0784
Db2= 0.0000
160TYR N
Db1= 0.0265
Db2= 0.2791
161VAL N
Db1= 0.0003
Db2= 0.2523
163ALA N
Db1= 0.0000
Db2= 0.1459
CSP [ppm]
Conc. Ligand [mM]

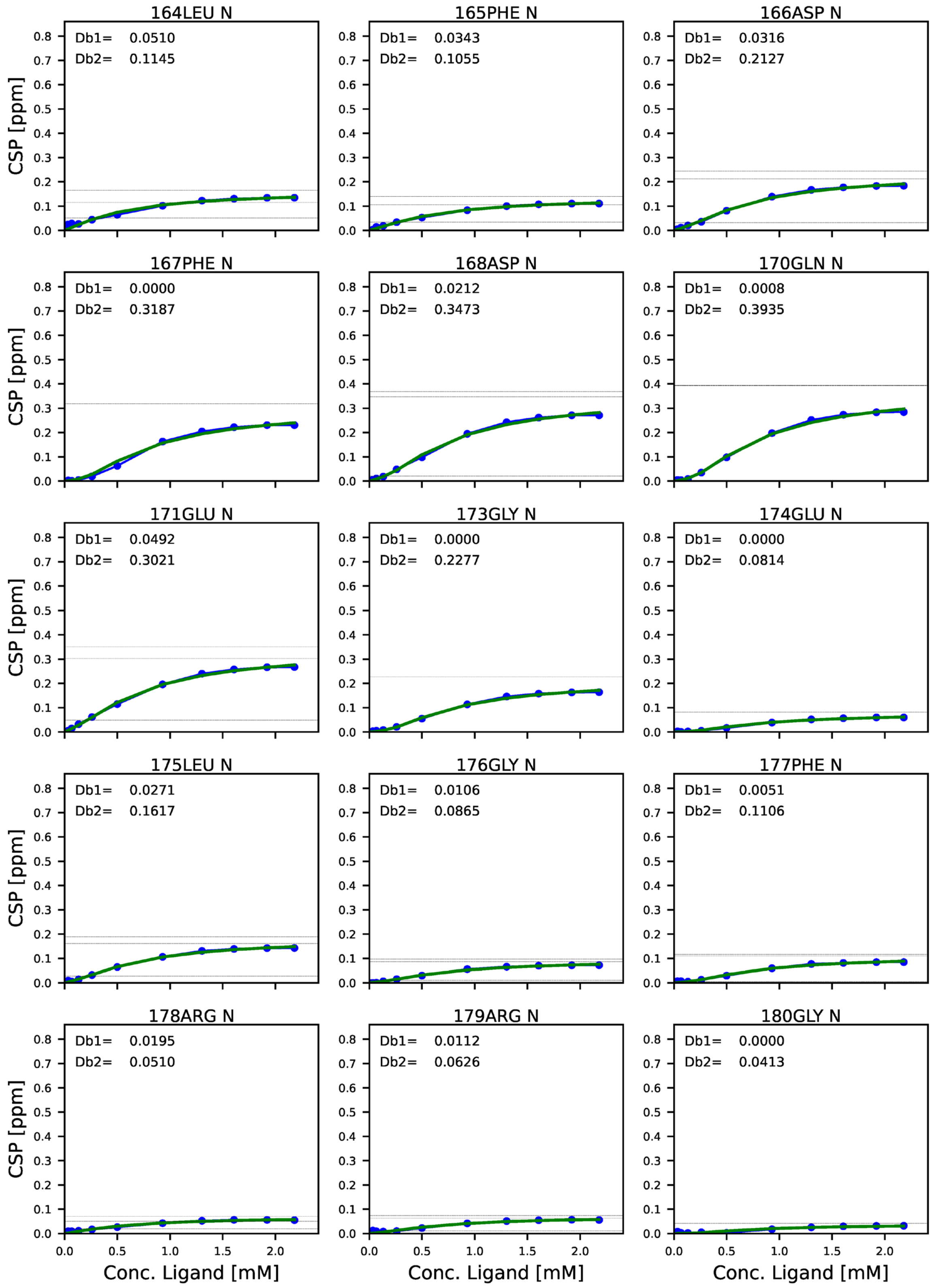
164LEU N
Db1= 0.0510
Db2= 0.1145
165PHE N
Db1= 0.0343
Db2= 0.1055
166ASP N
Db1= 0.0316
Db2= 0.2127
167PHE N
Db1= 0.0000
Db2= 0.3187
168ASP N
Db1= 0.0212
Db2= 0.3473
170GLN N
Db1= 0.0008
Db2= 0.3935
171GLU N
Db1= 0.0492
Db2= 0.3021
173GLY N
Db1= 0.0000
Db2= 0.2277
174GLU N
Db1= 0.0000
Db2= 0.0814
175LEU N
Db1= 0.0271
Db2= 0.1617
176GLY N
Db1= 0.0106
Db2= 0.0865
177PHE N
Db1= 0.0051
Db2= 0.1106
178ARG N
Db1= 0.0195
Db2= 0.0510
179ARG N
Db1= 0.0112
Db2= 0.0626
180GLY N
Db1= 0.0000
Db2= 0.0413
CSP [ppm]
Conc. Ligand [mM]

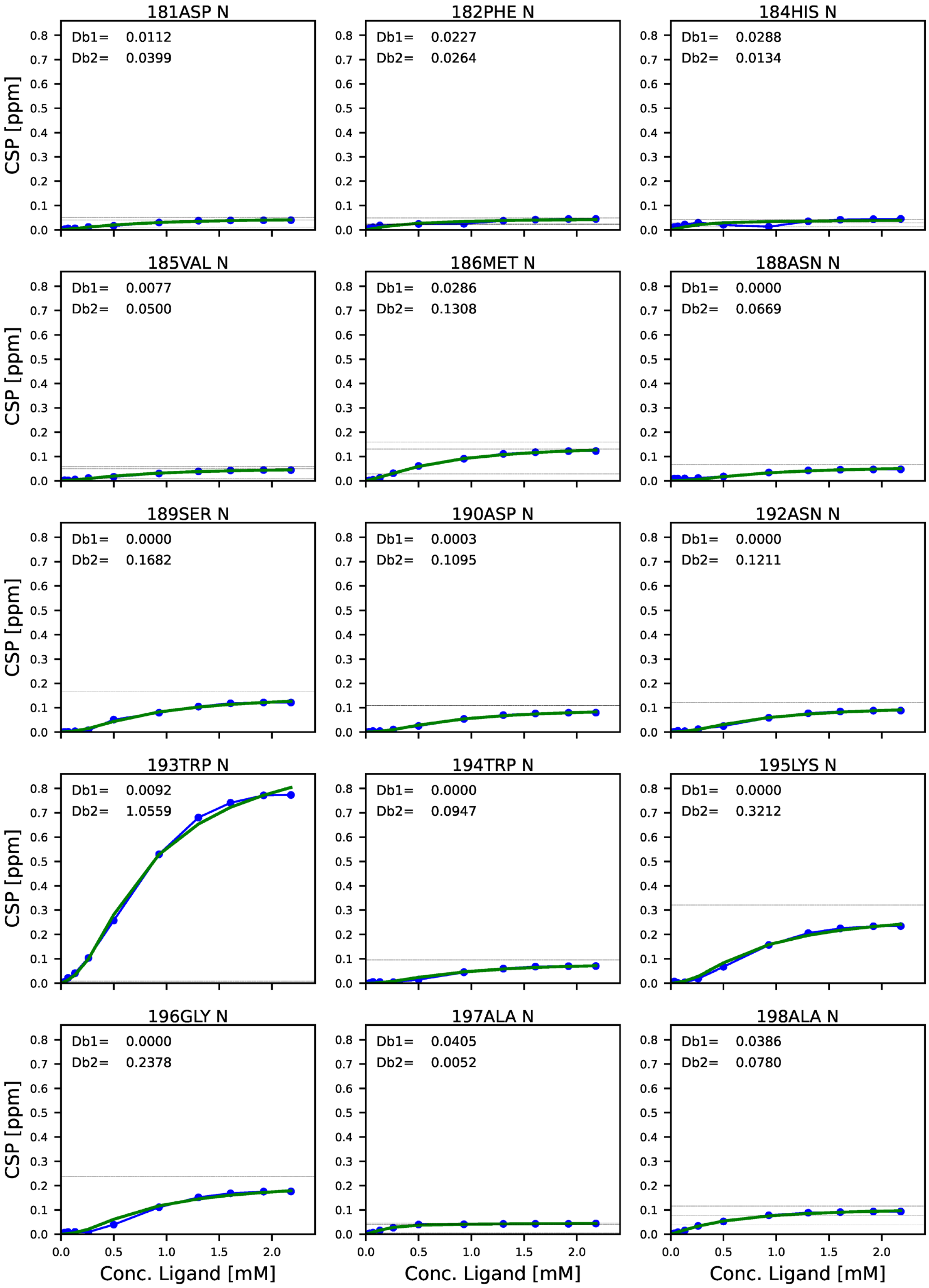
181ASP N
Db1= 0.0112
Db2= 0.0399
182PHE N
Db1= 0.0227
Db2= 0.0264
184HIS N
Db1= 0.0288
Db2= 0.0134
185VAL N
Db1= 0.0077
Db2= 0.0500
186MET N
Db1= 0.0286
Db2= 0.1308
188ASN N
Db1= 0.0000
Db2= 0.0669
189SER N
Db1= 0.0000
Db2= 0.1682
190ASP N
Db1= 0.0003
Db2= 0.1095
192ASN N
Db1= 0.0000
Db2= 0.1211
193TRP N
Db1= 0.0092
Db2= 1.0559
194TRP N
Db1= 0.0000
Db2= 0.0947
195LYS N
Db1= 0.0000
Db2= 0.3212
196GLY N
Db1= 0.0000
Db2= 0.2378
197ALA N
Db1= 0.0405
Db2= 0.0052
198ALA N
Db1= 0.0386
Db2= 0.0780
CSP [ppm]
Conc. Ligand [mM]

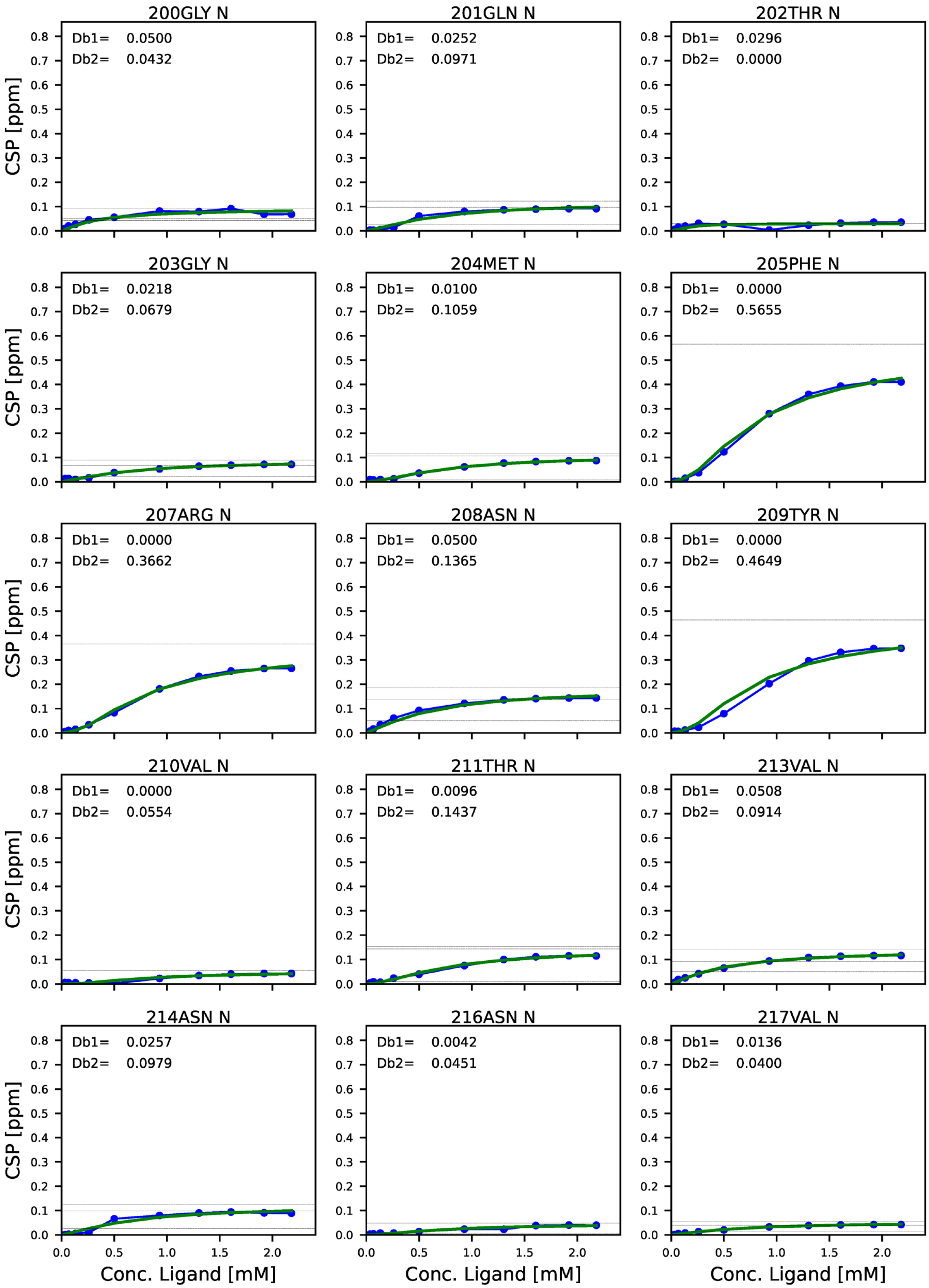
200GLY N
Db1= 0.0500
Db2= 0.0432
201GLN N
Db1= 0.0252
Db2= 0.0971
202THR N
Db1= 0.0296
Db2= 0.0000
203GLY N
Db1= 0.0218
Db2= 0.0679
204MET N
Db1= 0.0100
Db2= 0.1059
205PHE N
Db1= 0.0000
Db2= 0.5655
207ARG N
Db1= 0.0000
Db2= 0.3662
208ASN N
Db1= 0.0500
Db2= 0.1365
209TYR N
Db1= 0.0000
Db2= 0.4649
210VAL N
Db1= 0.0000
Db2= 0.0554
211THR N
Db1= 0.0096
Db2= 0.1437
213VAL N
Db1= 0.0508
Db2= 0.0914
214ASN N
Db1= 0.0257
Db2= 0.0979
216ASN N
Db1= 0.0042
Db2= 0.0451
217VAL N
Db1= 0.0136
Db2= 0.0400
CSP [ppm]
Conc. Ligand [mM]

Figure S11. Chemical shift perturbation profile mapped on each residue, against the concentration of ligand with SOS S4 PRM. The blue dots and lines indicate CSP values at each titration point, while the green lines represent the theoretical curves calculated based on the two-site independent binding model.

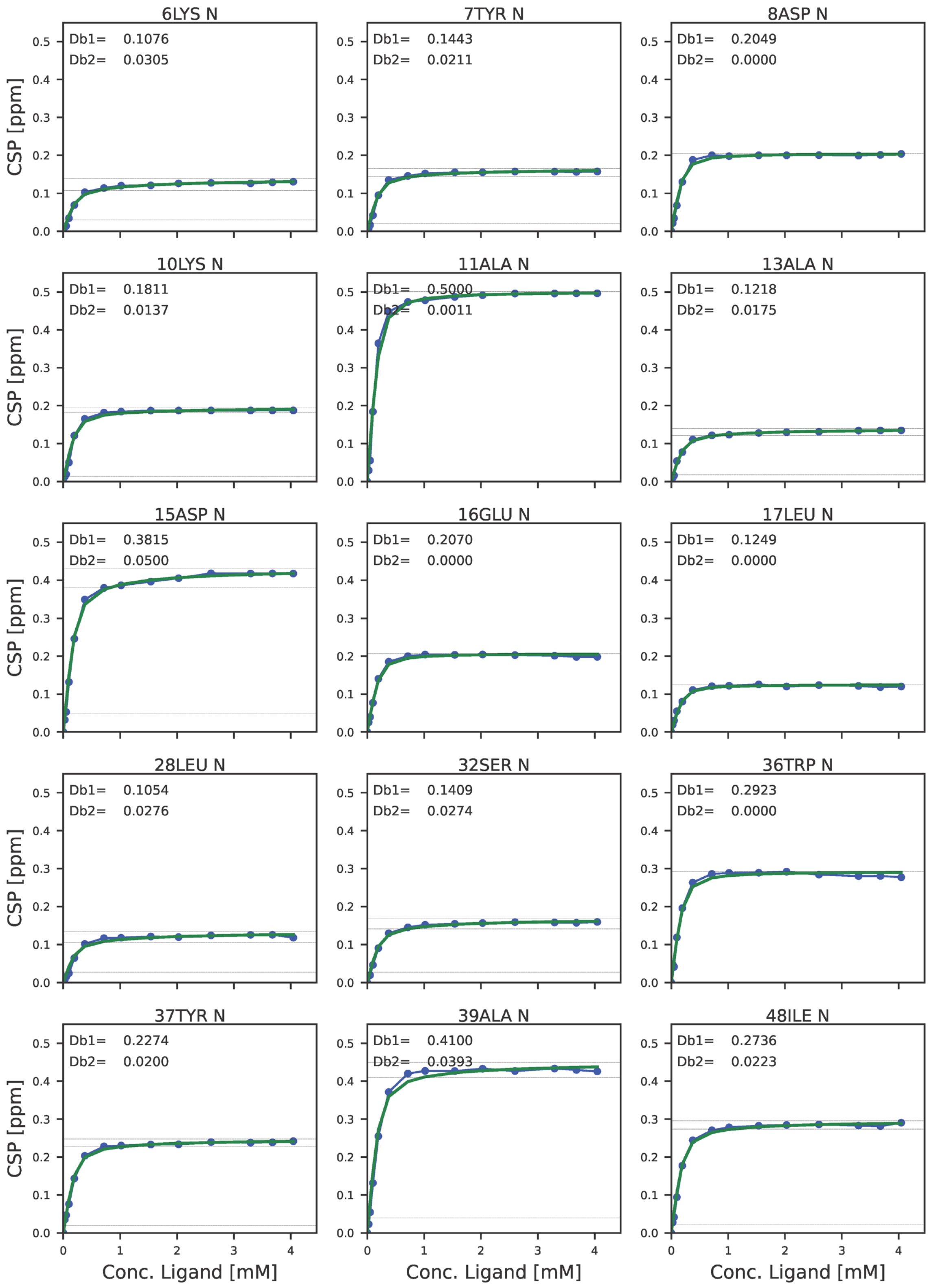
6LYS N
Db1= 0.1076
Db2= 0.0305
7TYR N
Db1= 0.1443
Db2= 0.0211
8ASP N
Db1= 0.2049
Db2= 0.0000
10LYS N
Db1= 0.1811
Db2= 0.0137
11ALA N
Db1= 0.5000
Db2= 0.0011
13ALA N
Db1= 0.1218
Db2= 0.0175
15ASP N
Db1= 0.3815
Db2= 0.0500
16GLU N
Db1= 0.2070
Db2= 0.0000
17LEU N
Db1= 0.1249
Db2= 0.0000
28LEU N
Db1= 0.1054
Db2= 0.0276
32SER N
Db1= 0.1409
Db2= 0.0274
36TRP N
Db1= 0.2923
Db2= 0.0000
37TYR N
Db1= 0.2274
Db2= 0.0200
39ALA N
Db1= 0.4100
Db2= 0.0393
48ILE N
Db1= 0.2736
Db2= 0.0223
CSP [ppm]
Conc. Ligand [mM]

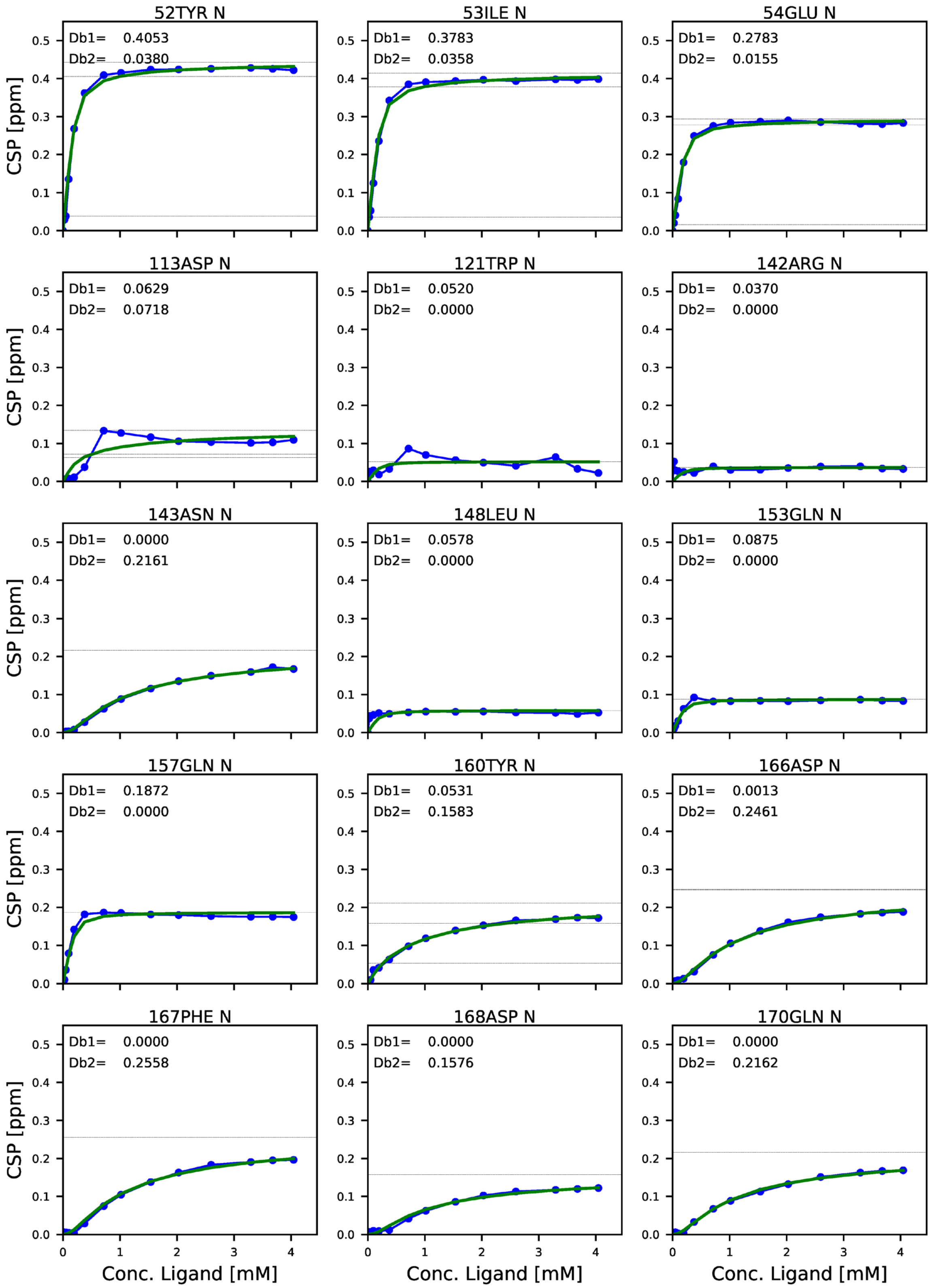
52TYR N
Db1= 0.4053
Db2= 0.0380
53ILE N
Db1= 0.3783
Db2= 0.0358
54GLU N
Db1= 0.2783
Db2= 0.0155
113ASP N
Db1= 0.0629
Db2= 0.0718
121TRP N
Db1= 0.0520
Db2= 0.0000
142ARG N
Db1= 0.0370
Db2= 0.0000
143ASN N
Db1= 0.0000
Db2= 0.2161
148LEU N
Db1= 0.0578
Db2= 0.0000
153GLN N
Db1= 0.0875
Db2= 0.0000
157GLN N
Db1= 0.1872
Db2= 0.0000
160TYR N
Db1= 0.0531
Db2= 0.1583
166ASP N
Db1= 0.0013
Db2= 0.2461
167PHE N
Db1= 0.0000
Db2= 0.2558
168ASP N
Db1= 0.0000
Db2= 0.1576
170GLN N
Db1= 0.0000
Db2= 0.2162
CSP [ppm]
Conc. Ligand [mM]

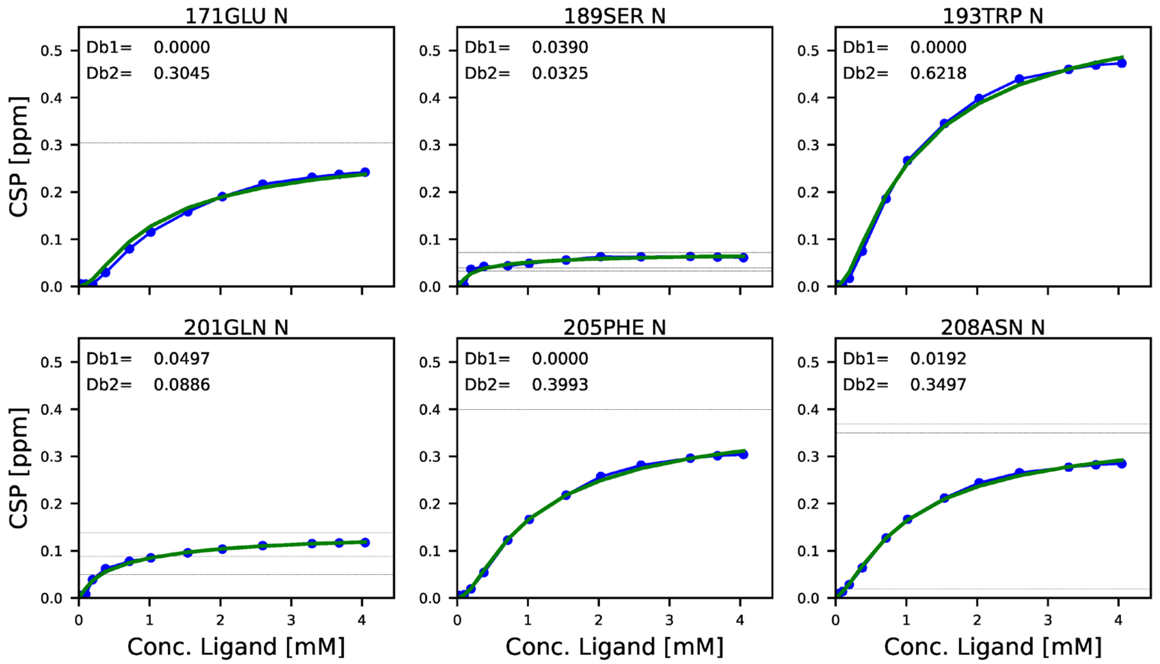

Figure S12. Chemical shift perturbation profile mapped on each residue, against the concentration of ligand with SOS S5 PRM. The blue dots and lines indicate CSP values at each titration point, while the green lines represent the theoretical curves calculated based on the two-site independent binding model.

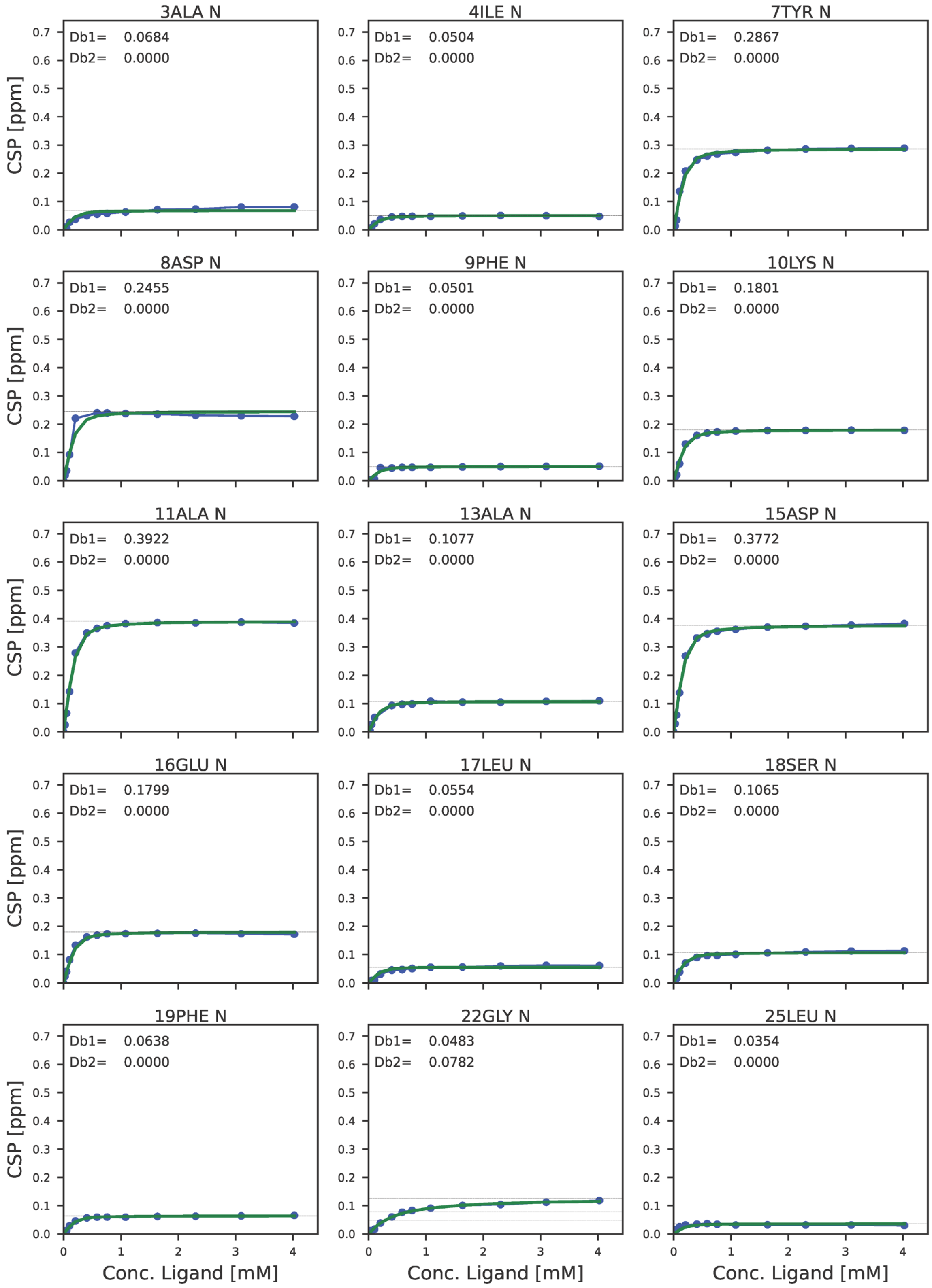
3ALA N
Db1= 0.0684
Db2= 0.0000
4ILE N
Db1= 0.0504
Db2= 0.0000
7TYR N
Db1= 0.2867
Db2= 0.0000
8ASP N
Db1= 0.2455
Db2= 0.0000
9PHE N
Db1= 0.0501
Db2= 0.0000
10LYS N
Db1= 0.1801
Db2= 0.0000
11ALA N
Db1= 0.3922
Db2= 0.0000
13ALA N
Db1= 0.1077
Db2= 0.0000
15ASP N
Db1= 0.3772
Db2= 0.0000
16GLU N
Db1= 0.1799
Db2= 0.0000
17LEU N
Db1= 0.0554
Db2= 0.0000
18SER N
Db1= 0.1065
Db2= 0.0000
19PHE N
Db1= 0.0638
Db2= 0.0000
22GLY N
Db1= 0.0483
Db2= 0.0782
25LEU N
Db1= 0.0354
Db2= 0.0000
CSP [ppm]
Conc. Ligand [mM]

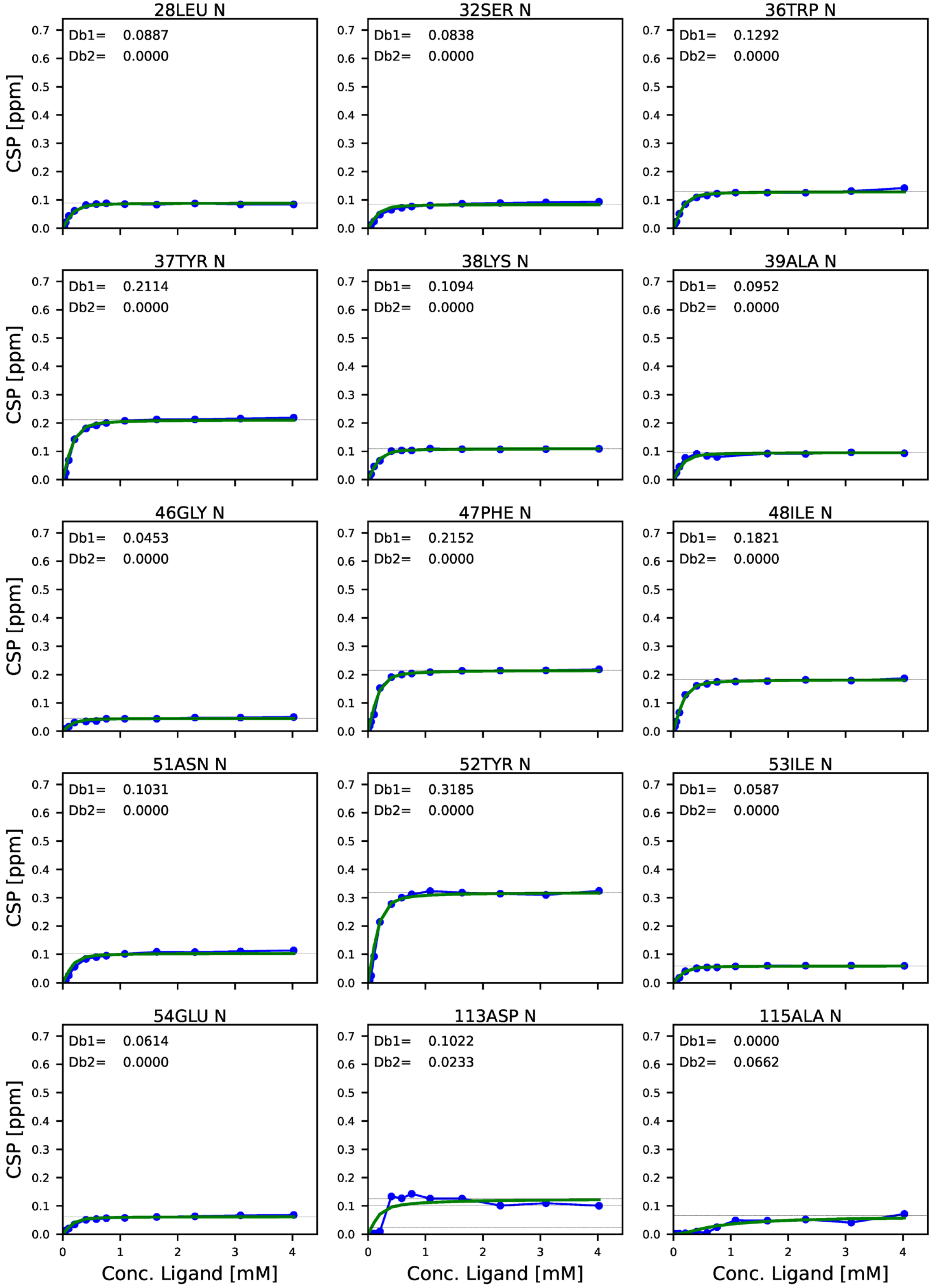
28LEU N
Db1= 0.0887
Db2= 0.0000
32SER N
Db1= 0.0838
Db2= 0.0000
36TRP N
Db1= 0.1292
Db2= 0.0000
37TYR N
Db1= 0.2114
Db2= 0.0000
38LYS N
Db1= 0.1094
Db2= 0.0000
39ALA N
Db1= 0.0952
Db2= 0.0000
46GLY N
Db1= 0.0453
Db2= 0.0000
47PHE N
Db1= 0.2152
Db2= 0.0000
48ILE N
Db1= 0.1821
Db2= 0.0000
51ASN N
Db1= 0.1031
Db2= 0.0000
52TYR N
Db1= 0.3185
Db2= 0.0000
53ILE N
Db1= 0.0587
Db2= 0.0000
54GLU N
Db1= 0.0614
Db2= 0.0000
113ASP N
Db1= 0.1022
Db2= 0.0233
115ALA N
Db1= 0.0000
Db2= 0.0662
CSP [ppm]
Conc. Ligand [mM]

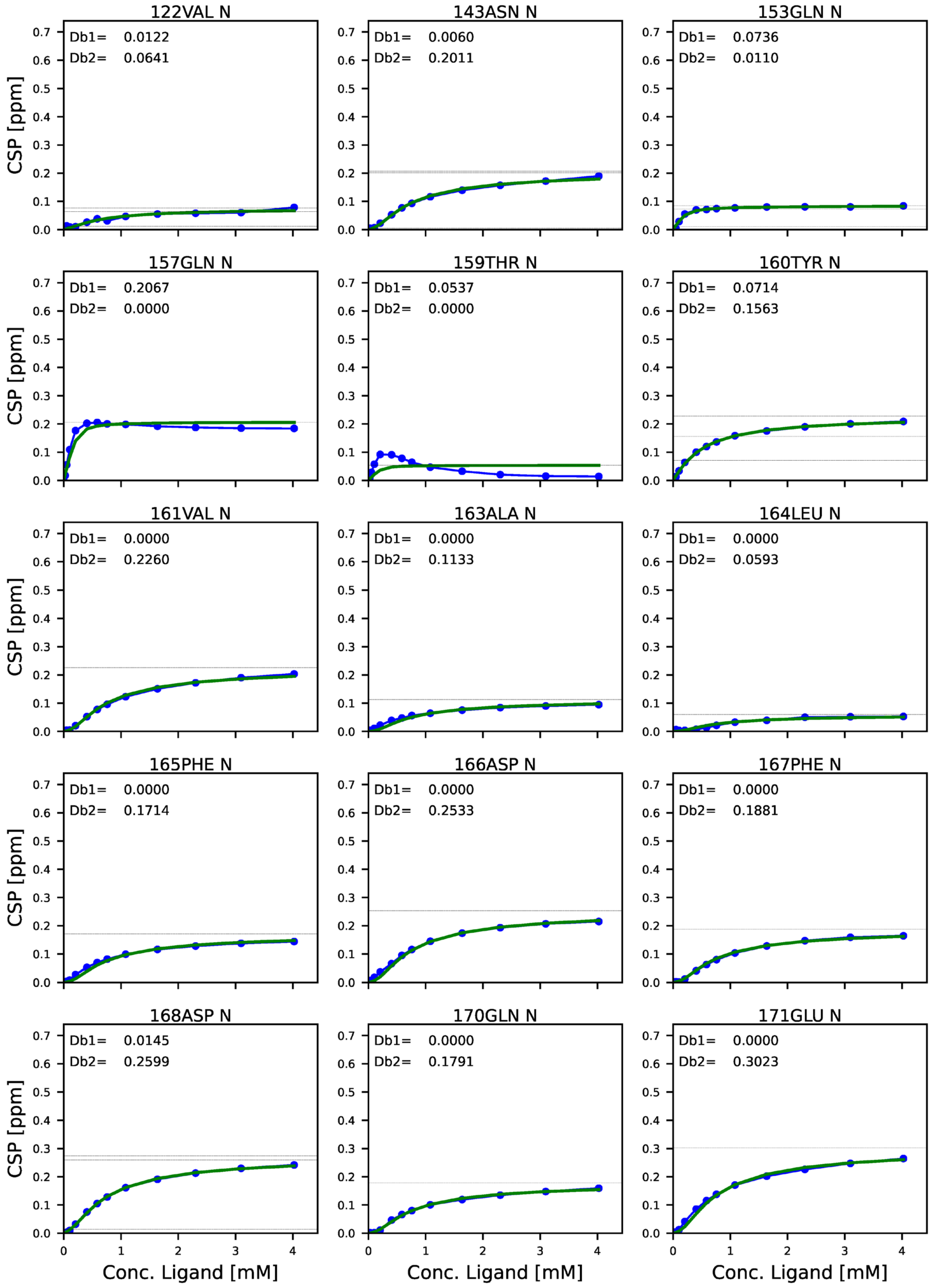
122VAL N
Db1= 0.0122
Db2= 0.0641
143ASN N
Db1= 0.0060
Db2= 0.2011
153GLN N
Db1= 0.0736
Db2= 0.0110
157GLN N
Db1= 0.2067
Db2= 0.0000
159THR N
Db1= 0.0537
Db2= 0.0000
160TYR N
Db1= 0.0714
Db2= 0.1563
161VAL N
Db1= 0.0000
Db2= 0.2260
163ALA N
Db1= 0.0000
Db2= 0.1133
164LEU N
Db1= 0.0000
Db2= 0.0593
165PHE N
Db1= 0.0000
Db2= 0.1714
166ASP N
Db1= 0.0000
Db2= 0.2533
167PHE N
Db1= 0.0000
Db2= 0.1881
168ASP N
Db1= 0.0145
Db2= 0.2599
170GLN N
Db1= 0.0000
Db2= 0.1791
171GLU N
Db1= 0.0000
Db2= 0.3023
CSP [ppm]
Conc. Ligand [mM]

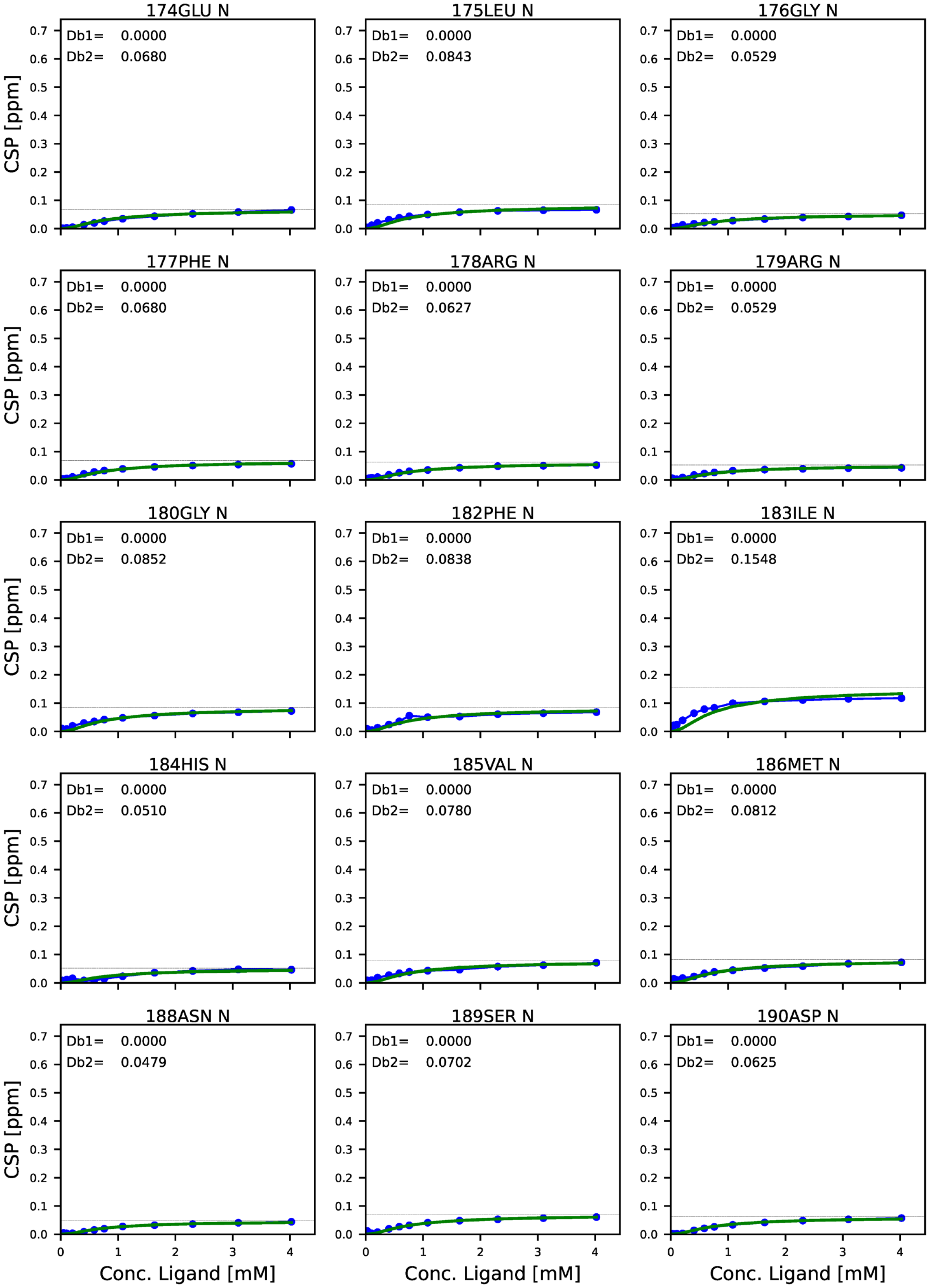
174GLU N
Db1= 0.0000
Db2= 0.0680
175LEU N
Db1= 0.0000
Db2= 0.0843
176GLY N
Db1= 0.0000
Db2= 0.0529
177PHE N
Db1= 0.0000
Db2= 0.0680
178ARG N
Db1= 0.0000
Db2= 0.0627
179ARG N
Db1= 0.0000
Db2= 0.0529
180GLY N
Db1= 0.0000
Db2= 0.0852
182PHE N
Db1= 0.0000
Db2= 0.0838
183ILE N
Db1= 0.0000
Db2= 0.1548
184HIS N
Db1= 0.0000
Db2= 0.0510
185VAL N
Db1= 0.0000
Db2= 0.0780
186MET N
Db1= 0.0000
Db2= 0.0812
188ASN N
Db1= 0.0000
Db2= 0.0479
189SER N
Db1= 0.0000
Db2= 0.0702
190ASP N
Db1= 0.0000
Db2= 0.0625
CSP [ppm]
Conc. Ligand [mM]

Figure S13. Chemical shift perturbation profile mapped on each residue, against the concentration of ligand with SOS S9 PRM. The blue dots and lines indicate CSP values at each titration point, while the green lines represent the theoretical curves calculated based on the two-site independent binding model.

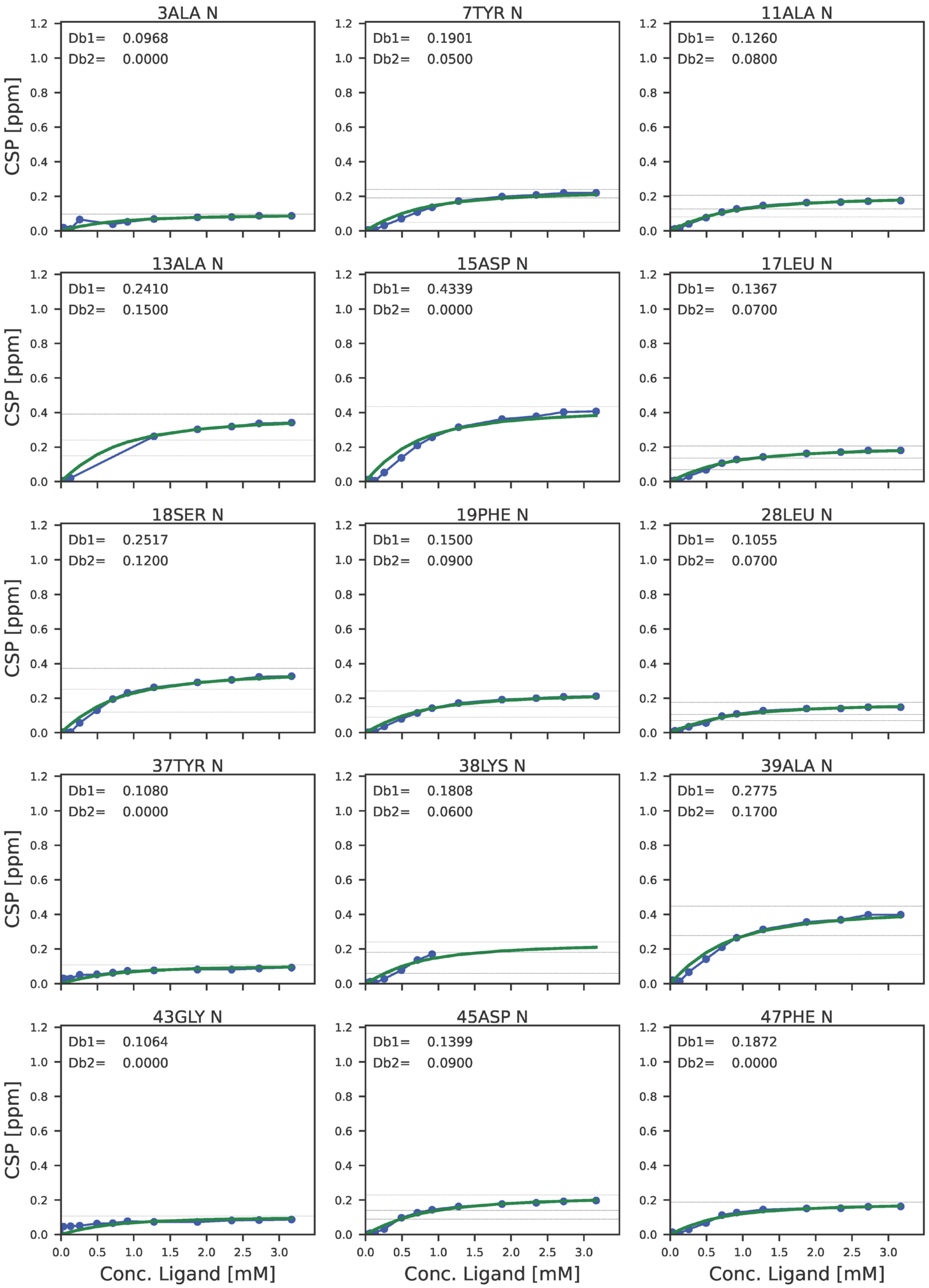
3ALA N
Db1= 0.0968
Db2= 0.0000
7TYR N
Db1= 0.1901
Db2= 0.0500
11ALA N
Db1= 0.1260
Db2= 0.0800
13ALA N
Db1= 0.2410
Db2= 0.1500
15ASP N
Db1= 0.4339
Db2= 0.0000
17LEU N
Db1= 0.1367
Db2= 0.0700
18SER N
Db1= 0.2517
Db2= 0.1200
19PHE N
Db1= 0.1500
Db2= 0.0900
28LEU N
Db1= 0.1055
Db2= 0.0700
37TYR N
Db1= 0.1080
Db2= 0.0000
38LYS N
Db1= 0.1808
Db2= 0.0600
39ALA N
Db1= 0.2775
Db2= 0.1700
43GLY N
Db1= 0.1064
Db2= 0.0000
45ASP N
Db1= 0.1399
Db2= 0.0900
47PHE N
Db1= 0.1872
Db2= 0.0000
CSP [ppm]
Conc. Ligand [mM]

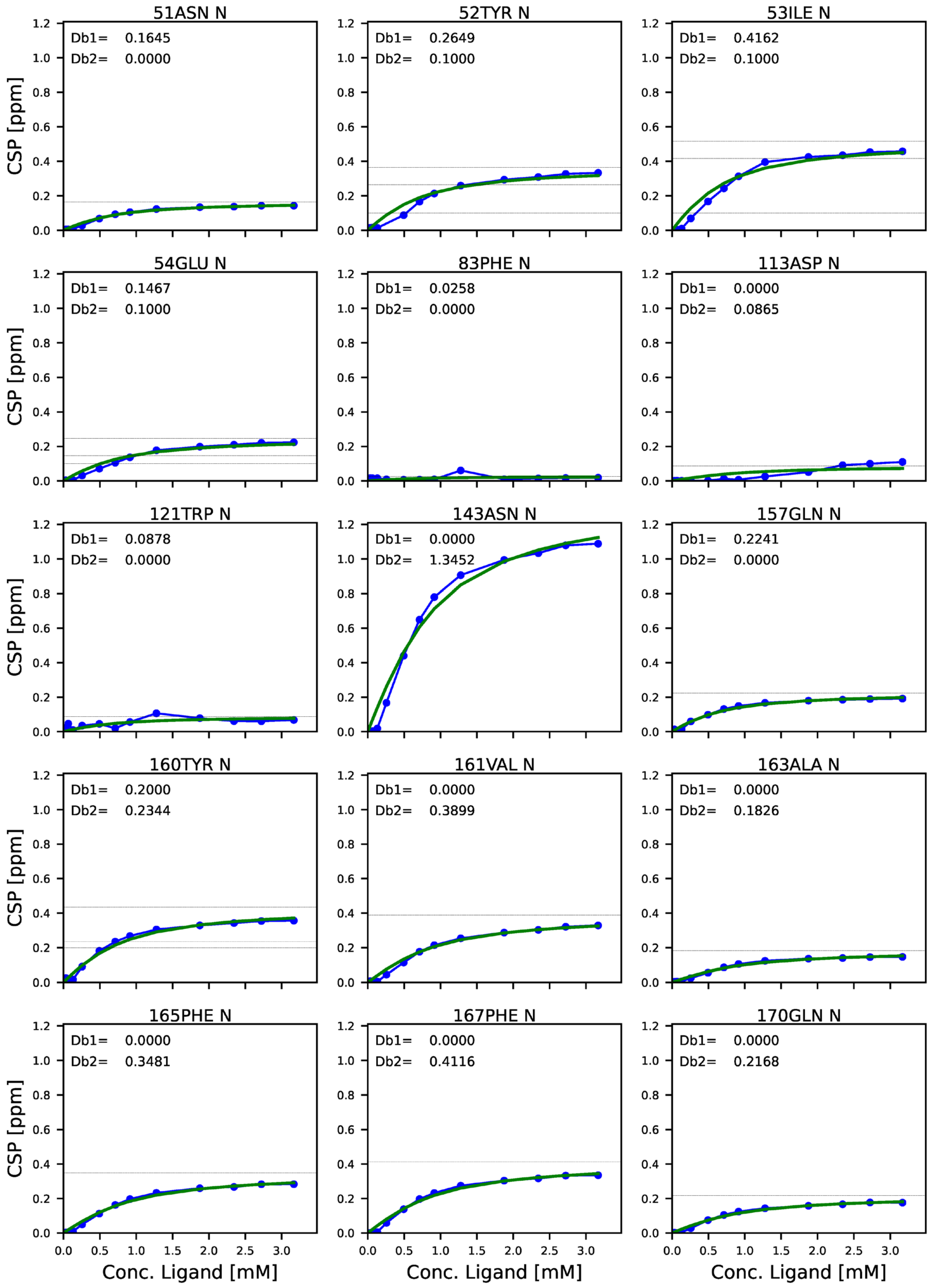
51ASN N
Db1= 0.1645
Db2= 0.0000
52TYR N
Db1= 0.2649
Db2= 0.1000
53ILE N
Db1= 0.4162
Db2= 0.1000
54GLU N
Db1= 0.1467
Db2= 0.1000
83PHE N
Db1= 0.0258
Db2= 0.0000
113ASP N
Db1= 0.0000
Db2= 0.0865
121TRP N
Db1= 0.0878
Db2= 0.0000
143ASN N
Db1= 0.0000
Db2= 1.3452
157GLN N
Db1= 0.2241
Db2= 0.0000
160TYR N
Db1= 0.2000
Db2= 0.2344
161VAL N
Db1= 0.0000
Db2= 0.3899
163ALA N
Db1= 0.0000
Db2= 0.1826
165PHE N
Db1= 0.0000
Db2= 0.3481
167PHE N
Db1= 0.0000
Db2= 0.4116
170GLN N
Db1= 0.0000
Db2= 0.2168
CSP [ppm]
Conc. Ligand [mM]

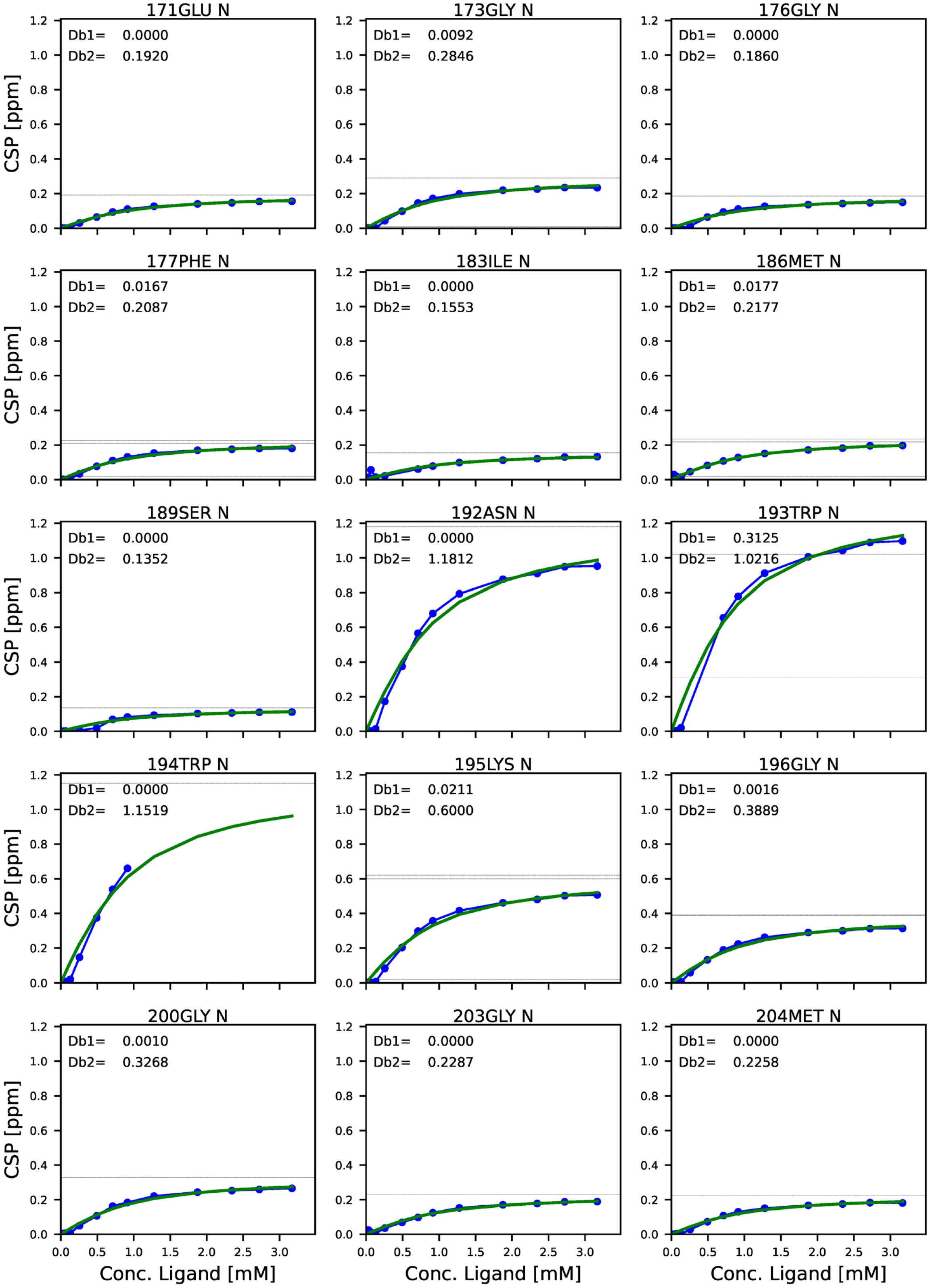
171GLU N
Db1= 0.0000
Db2= 0.1920
173GLY N
Db1= 0.0092
Db2= 0.2846
176GLY N
Db1= 0.0000
Db2= 0.1860
177PHE N
Db1= 0.0167
Db2= 0.2087
183ILE N
Db1= 0.0000
Db2= 0.1553
186MET N
Db1= 0.0177
Db2= 0.2177
189SER N
Db1= 0.0000
Db2= 0.1352
192ASN N
Db1= 0.0000
Db2= 1.1812
193TRP N
Db1= 0.3125
Db2= 1.0216
194TRP N
Db1= 0.0000
Db2= 1.1519
195LYS N
Db1= 0.0211
Db2= 0.6000
196GLY N
Db1= 0.0016
Db2= 0.3889
200GLY N
Db1= 0.0010
Db2= 0.3268
203GLY N
Db1= 0.0000
Db2= 0.2287
204MET N
Db1= 0.0000
Db2= 0.2258
CSP [ppm]
Conc. Ligand [mM]

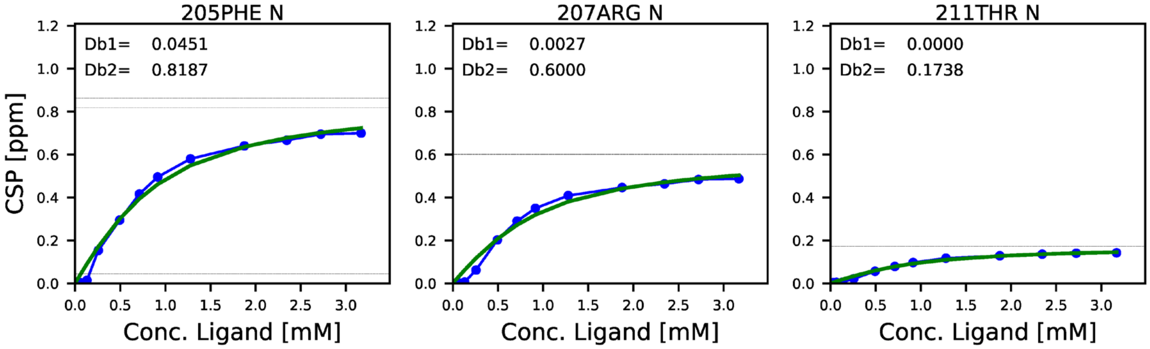

Figure S14. Chemical shift perturbation profile mapped on each residue, against the concentration of ligand with SOS S10 PRM. The blue dots and lines indicate CSP values at each titration point, while the green lines represent the theoretical curves calculated based on the two-site independent binding model.

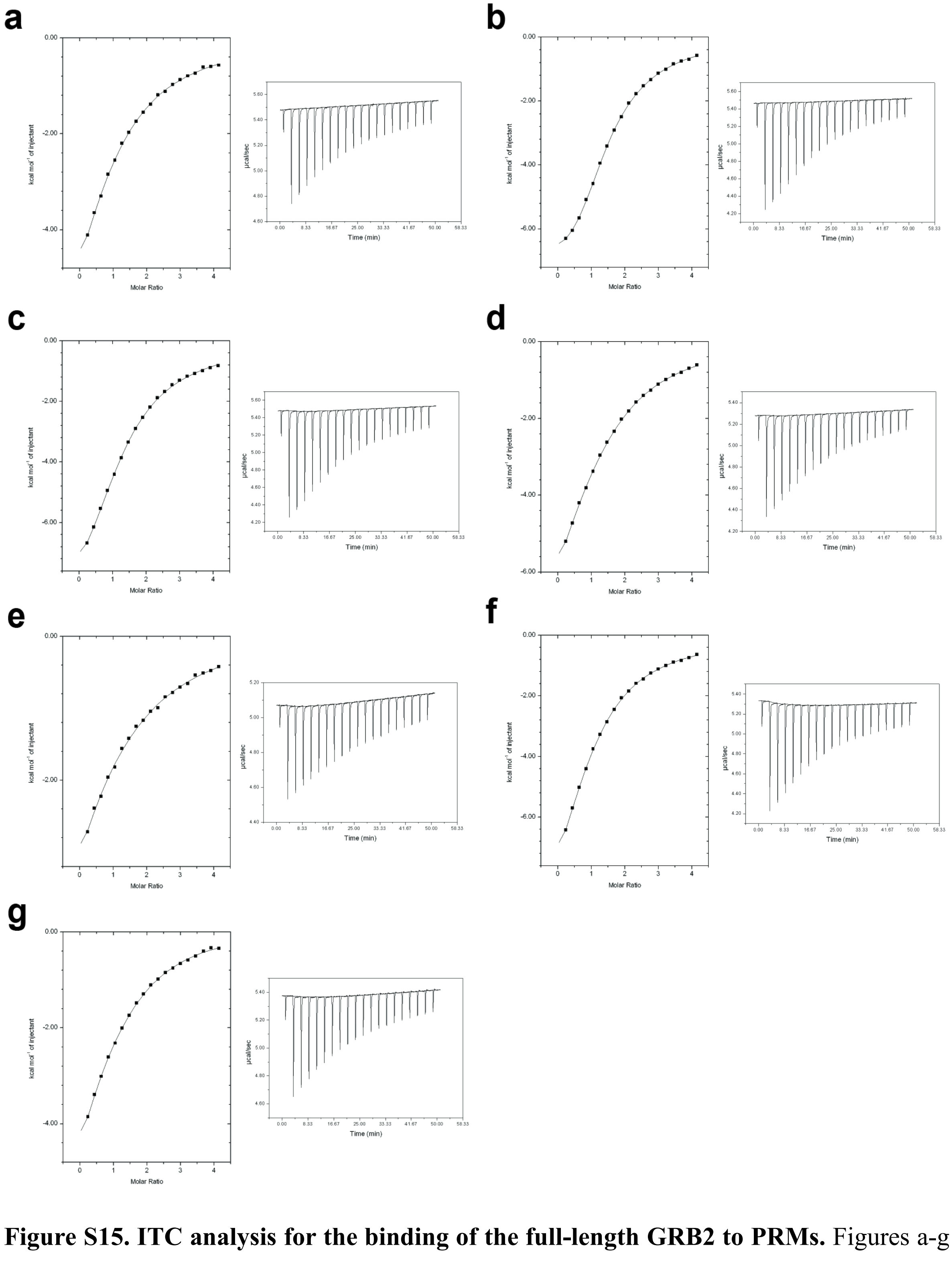

Figure S15. ITC analysis for the binding of the full-length GRB2 to PRMs. Figures a-g indicate ITC analysis for S2 (**a**), S4 (**b**), S5 (**c**), S6 (**d**), S8 (**e**) S9 (**f**), and S10 (**g**) PRMs,

respectively. Data with S1, S3, and S7 are not shown here because of no clear thermogram curves. The solid lines show the fit of the data to a "Two Set of Sites" model based on the binding of a ligand to a macromolecule, as incorporated in the Origin software.

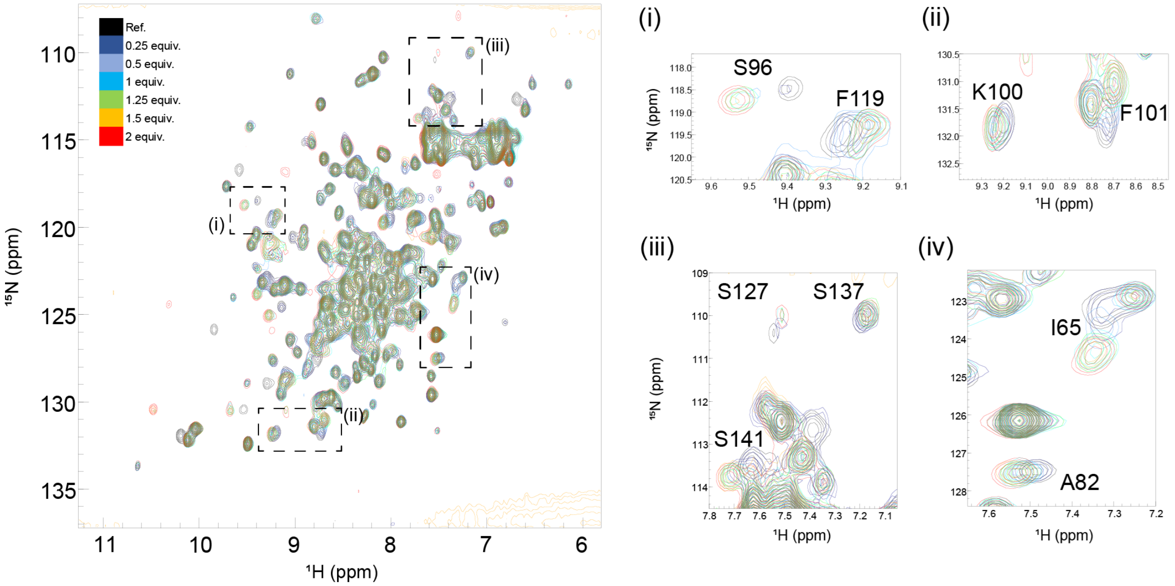

Figure S16. Overlays of 2D ^{1}H-^{15}N HSQC spectra from multipoint titrations of ^{15}N-labelled GRB2 exclusively with the EGFR phosphorylated peptide (EpYINSQV). The peptide concentration was increased stepwise (the protein: peptide molar ratio of 1:0.25, 1:0.5, 1:1, 1:1.25, 1:1.5, and 1:2). In this figure, the colour codes of ^{1}H-^{15}N correlation cross-peaks at each titration point, showing the molar ratio of GRB2: EGFR, are as follows: black (1:0); green (1:0.25); blue (1:0.5); yellow (1:0.75); magenta (1:1); cyan (1:1.25); brown (1:1.5); coral (1:2); red (1:4). Cross peaks that showed large chemical shift changes were annotated.

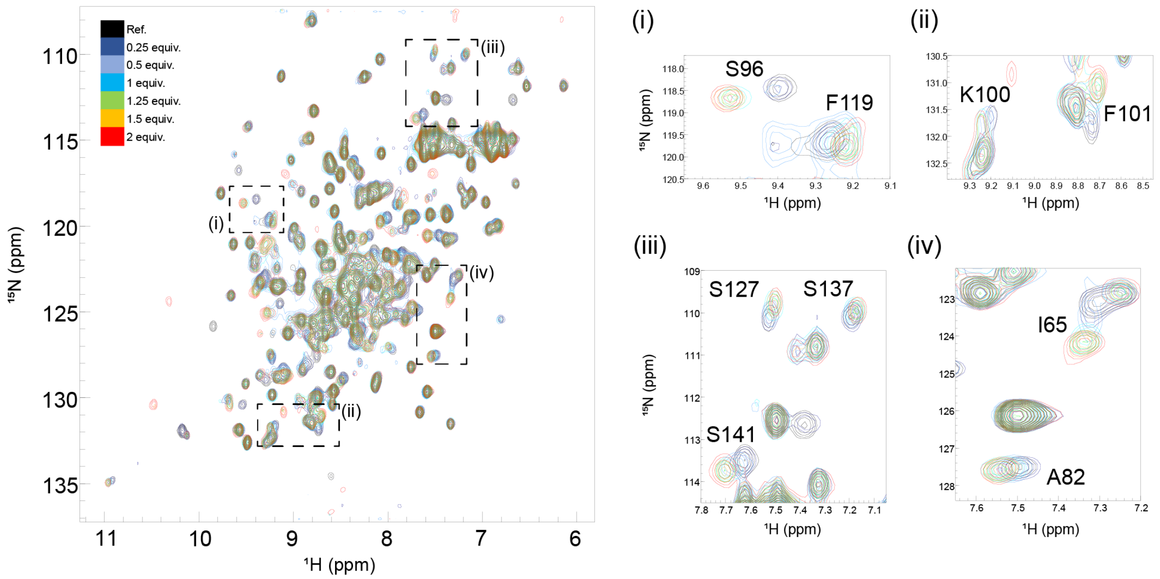

Figure S17. Overlays of 2D ^{1}H-^{15}N HSQC spectra from multipoint titrations of ^{15}N-labelled GRB2 with the EGFR phosphorylated peptide (EpYINSQV), in the presence of S4 PRM. The peptide concentration was increased stepwise (the protein: peptide molar ratio of 1:0.25, 1:0.5, 1:1, 1:1.25, 1:1.5, and 1:2). In this figure, the colour codes of ^{1}H-^{15}N correlation cross-peaks at each titration point, showing the molar ratio of GRB2: EGFR, are as follows: black (1:0); green (1:0.25); blue (1:0.5); yellow (1:0.75); magenta (1:1); cyan (1:1.25); brown (1:1.5); coral (1:2); red (1:4). Cross peaks that showed large chemical shift changes were annotated.

Table S1. Dissociation constants derived from ITC analysis for the full length GRB3 and isolated SH3 domains against SOS1 PRMs

		ITC analysis			
PRM	Sequence	K_D (μM) from the full length GRB2[a]		K_D (μM) from isolated SH3[b]	
		K_{D1}	K_{D2}	K_{D1}	K_{D2}
S1	VPPPVPPRRR (1148-1159)	NA	NA	—	—
S2	VPPPVPPRRR (1148-1159)	65.1 ± 23.7	NA	—	—
S3	VPPPVPPRRR (1148-1159)	NA	NA	—	—
S4	VPPPVPPRRR (1148-1159)	5.3 ± 0.4	51.3 ± 1.7	39 ± 1	125 ± 13
S5	DSPPAOPPRQPT (1177-1188)	15.9 ± 1.5	109.7 ± 6.5	56 ± 5	1396 ± 87
S6	DSPPAOPPRQPT (1177-1188)	40.1 ± 6.5	97.3 ± 10.5	117 ± 2	1718 ± 33
S7	DSPPAOPPRQPT (1177-1188)	NA	NA	—	—
S8	DSPPAOPPRQPT (1177-1188)	122.9 ± 18.6	NA	—	—
S9	IAGPPVPPRQST (1287-1298)	35.6 ± 4.2	640.0 ± 291.1	82 ± 1	1318 ± 44
S10	PKLPPKTYKREH (1303-1314)	71.5 ± 24.2	130.8 ± 54.4	—	—

[a]The dissociation constants were obtained by globally fitting ITC data to the "Two Set of Sites" model using MicroCal ITC-ORIGIN Analysis Software (Malvern Panalytical). [b]The dissociation constants are published data from ITC in the previous work using isolated SH3 domains{McDonald, 2009 #67}.
